# Thyroglobulin Interactome Profiling Defines Altered Proteostasis Topology Associated With Thyroid Dyshormonogenesis

**DOI:** 10.1074/mcp.RA120.002168

**Published:** 2020-12-08

**Authors:** Madison T. Wright, Logan Kouba, Lars Plate

**Affiliations:** 1 Department of Chemistry, Vanderbilt University, Nashville, Tennessee, USA; 2 Department of Biological Sciences, Vanderbilt University, Nashville, Tennessee, USA

**Keywords:** affinity purification - mass spectrometry, protein folding, protein-protein interactions, protein quality control, cell secretion, congenital hypothyroidism, tandem mass tags, BiP, binding immunoglobulin protein, CALR, calreticulin, CANX, calnexin, CH, congenital hypothyroidism, ChEL, cholinesterase-like domain, CHX, cycloheximide, DNAJB11, DnaJ heat shock protein family member B11, DNAJC10, DnaJ heat shock protein family member C10, DSP, dithiobis(succinimidyl propionate), EDEM, ER degradation enhancing alpha-mannosidase, EndoH, endoglycosidase H, ER, endoplasmic reticulum, ERAD, ER-associated degradation, FOXRED2, RAD-dependent oxidoreductase domain containing 2, GANAB, glucosidase II alpha subunit, GO, gene ontology, GRP94, 94 kDa glucose regulated protein, Hsp70/40, heat shock protein 70 kDa and 40 kDa chaperone/co-chaperone system, Hsp90, heat shock protein 90, MAN1B1, mannosidase alpha class 1B member 1, N-glycosylation, asparagine-linked protein glycosylation, OS-9, OS9 endoplasmic reticulum lectin, OST, oligosaccharyl transferase complex, PDIA3, protein disulfide isomerase family A member 3, PDIA4, protein disulfide isomerase family A member 4, PDIA6, protein disulfide isomerase family A member 6, PN, proteostasis network, PQC, protein quality control, STT3A, oligosaccharyl transferase complex catalytic subunit A, STT3B, oligosaccharyl transferase complex catalytic subunit B, SEL1L, SEL1L adaptor subunit of ERAD E3 ubiquitin ligase, T3, Triiodothyronine, T4, thyroxine, Tg, thyroglobulin, TMT, tandem mass tags, UGGT1, UDP-glucose glycoprotein glucosyltansferase 1, UPR, unfolded protein response

## Abstract

Thyroglobulin (Tg) is a secreted iodoglycoprotein serving as the precursor for triiodothyronine and thyroxine hormones. Many characterized Tg gene mutations produce secretion-defective variants resulting in congenital hypothyroidism. Tg processing and secretion is controlled by extensive interactions with chaperone, trafficking, and degradation factors comprising the secretory proteostasis network. While dependencies on individual proteostasis network components are known, the integration of proteostasis pathways mediating Tg protein quality control and the molecular basis of mutant Tg misprocessing remain poorly understood. We employ a multiplexed quantitative affinity purification–mass spectrometry approach to define the Tg proteostasis interactome and changes between WT and several congenital hypothyroidism variants. Mutant Tg processing is associated with common imbalances in proteostasis engagement including increased chaperoning, oxidative folding, and engagement by targeting factors for endoplasmic reticulum–associated degradation. Furthermore, we reveal mutation-specific changes in engagement with N-glycosylation components, suggesting distinct requirements for 1 Tg variant on dual engagement of both oligosaccharyltransferase complex isoforms for degradation. Modulating dysregulated proteostasis components and pathways may serve as a therapeutic strategy to restore Tg secretion and thyroid hormone biosynthesis.

## Highlights

• Quantitative multiplexed affinity purification—mass spectrometry was used to map the comprehensive thyroglobulin interactome.
• Tg interactome consist of a diverse set of protein quality control components.
• Mutants share imbalances with folding, disulfide processing, and degradation elements.
• N-glycosylation plays a key role in the degradation of mutant thyroglobulin.

## Graphical Abstract

**Figure:**
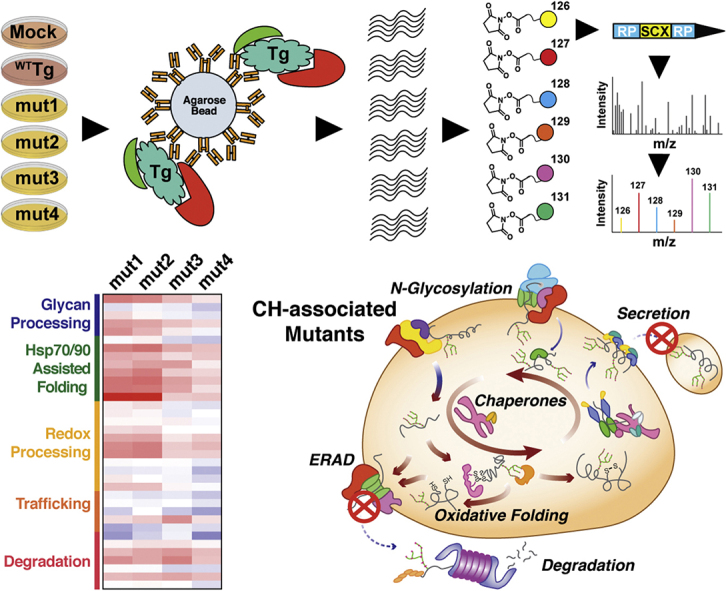

## In Brief

Congenital hypothyroidism can result from mutations in the thyroglobulin (Tg) gene impairing protein secretion of the defective Tg variants. The molecular basis of mutant Tg misprocessing has remained poorly understood. Here we map the Tg interactome and quantify mutation specific changes in protein–protein interactions between Tg and protein quality control components. By integrating interactions across protein quality control pathways, we highlight shared imbalances in protein folding, disulfide processing, and degradation, and reveal mutant-specific roles for N-glycosylation in Tg processing.

**Fig. 1:**
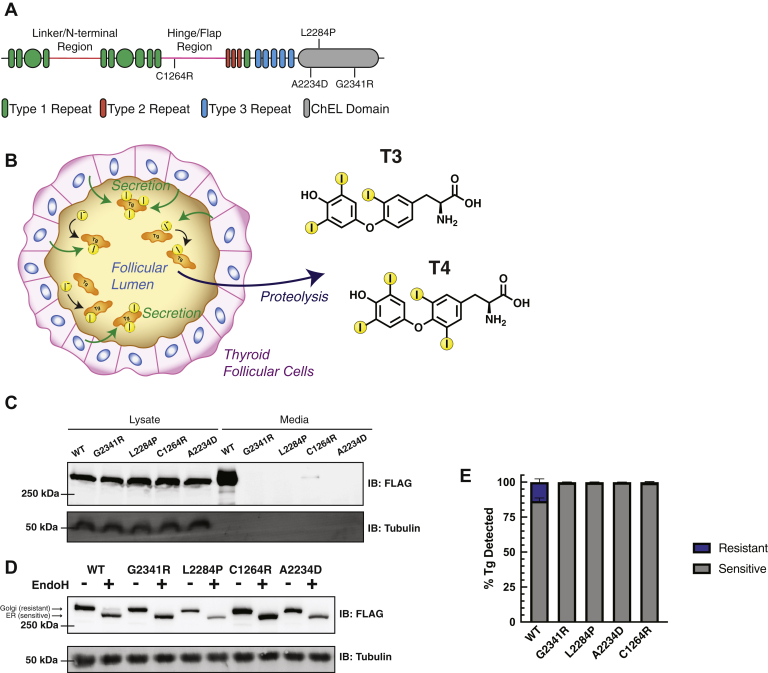
**Distinct Tg mutants present secretion defects.***A*, schematic of Tg domain organization consisting of cysteine rich repeats, a linker/N-terminal region, and hinge/flap region followed by a cholinesterase-like (ChEL) C-terminal domain. *B*, schematic detailing Tg processing and subsequent hormone production. Tg is synthesized in follicular cells and secreted into the follicular lumen where it undergoes iodination and is stored. Tg is later taken up and proteolyzed leading to the liberation of T3 and T4 hormones. *C*, immunoblot for Flag-tagged Tg expressed in transiently transfected HEK293T cells. All Tg variants are detected in the lysate while only WT is detected in cell culture media. *D*, Western blot for Tg probing EndoH sensitivity to remove high-mannose glycans of ER-localized Tg. Golgi-associated (resistant) and ER-associated (sensitive) bands are indicated by arrows. Only WT Tg develops EndoH resistance, indicating it is able to traverse the medial Golgi. All CH-associated Tg mutants are EndoH sensitive and unable to reach the medial Golgi. *E*, quantification of EndoH sensitivity in (*D*). All Tg mutants are 100% EndoH sensitive, showing they are retained within the ER, unable to reach the medial Golgi, and thus model a hypothyroidism phenotype. Error bars show SEM for four biological replicates. CH, congenital hypothyroidism; Tg, thyroglobulin; ER, endoplasmic reticulum.

Thyroid hormone biosynthesis is an intricate and multifaceted process involving a sequence of biochemical reactions (1, 2, 3, 4). Triiodothyronine (T3) and thyroxine (T4) hormones are necessary for normal growth and development in utero and early childhood and go on to regulate primary metabolism in adulthood (5, 6). Hypothyroidism and dyshormonogenesis stemming from mutations or damage to the biosynthetic components ultimately results in decreased or complete loss in production of T3 and T4. Congenital hypothyroidism (CH) affects approximately 1:2000 to 1:4000 newborns and if not detected and addressed can lead to severe and permanent neurological damage, including mental retardation (7, 8). A critical gene involved in thyroid hormone biosynthesis and CH pathology is thyroglobulin (Tg), encoding the prohormone protein for T3 and T4 (Fig. 1*A*). There are 167 documented Tg mutations that impair proper production, folding, or processing leading to dyshormonogenesis (5). Missense mutations resulting in full-length but folding-incompetent Tg disrupt normal protein homeostasis (proteostasis) and lead to decreased or complete loss of Tg protein secretion into the thyroid follicular lumen, a key step in hormone production. Instead, mutant Tg variants accumulate within the endoplasmic reticulum (ER) of thyroid follicular cells (9, 10).

While many CH-associated folding-incompetent Tg mutations have been documented, the molecular mechanisms of Tg folding and processing controlled by the proteostasis network (PN), consisting of chaperones, co-chaperones, folding enzymes, trafficking factors, and degradation factors, remain incompletely understood. Coordination of these PN components ensures the proper folding, trafficking, and degradation of clients such as Tg through a process cumulatively referred to as protein quality control (PQC) (11, 12, 13). Previous studies have shown that CH-associated Tg mutants exhibit increased interactions with individual PN components including binding immunoglobulin protein (BiP), 94 kDa glucose-regulated protein (GRP94), protein disulfide isomerase family A member 3 (PDIA3), calnexin (CANX), and calreticulin (CALR) that aid in folding and processing (14, 15, 16, 17, 18, 19, 20). Nonetheless, it remains unclear how these PN components and their respective subnetworks cooperate to mediate proper Tg folding and secretion. Furthermore, identifying which of these components or subnetworks are implicated in the improper processing of mutant Tg has remained elusive. The current collection of known interactors, identified through traditional immunoprecipitation and immunoblotting strategies, is likely limited as these methods are not conducive to discovery-based investigations. Additionally, little work has focused on characterizing mutation-specific changes in PN engagement. No disease modifying therapies currently exist to restore secretion of destabilized Tg, but devising such strategies would be particularly critical considering the increased prevalence of dyshormonogenesis among newborns and complications arising from the current “gold standard” of hormone therapy treatments in the clinic (7, 21). Identifying the complete Tg interactome and defining the molecular mechanisms of altered PN engagement for mutant Tg variants may reveal areas of PQC that can be targeted therapeutically to rescue the secretion of these CH-associated variants. Modulation of individual PN components or entire pathways has shown recent promise as a therapeutic strategy to combat a number of protein folding diseases, including light-chain amyloidosis, transthyretin amyloidosis, and polyglutamine-associated neuropathies (22, 23, 24, 25).

Here, we present a tandem mass tag-based quantitative interactome proteomics method that allowed us to globally profile several CH-associated mutant Tg variants. Compared with other interactome studies (26, 27, 28), the multiplexing capabilities enable a head-to-head comparative analysis of five distinct protein variants. While chaperone complexes and client recognition for select chaperones have been mapped (29, 30, 31), system-wide investigations into PN processing of individual client proteins are lacking. The current study describes the identification of a comprehensive PN interactome for WT Tg and several secretion-deficient mutant variants. Comparison of the PN interactome for the CH-associated mutant variants to WT Tg allowed us to gain mechanistic insights into shared PQC defects that are responsible for the loss of secretion of all destabilized variants. Our data support a model whereby the destabilized Tg variants are retained intracellularly through increased interactions with chaperoning and oxidative protein folding pathway components. We also find evidence that Tg mutants are increasingly routed toward ER-associated degradation (ERAD) yet exhibit overall lower engagement with proteasomal degradation machinery. Furthermore, we find mutation-specific interactome remodeling with components of N-glycosylation and ER lectin protein folding components. Mutant-specific interaction changes suggest that such Tg variants have distinct imbalances associated with their aberrant folding and processing within the ER, leading to the loss of secretion.

## Experimental Procedures

### Plasmids and Antibodies

FLAG-tagged Tg in pcDNA3.1+/C-(K)-DYK plasmid was purchased from Genscript (Clone ID OHu20241). Site-directed mutagenesis was then performed to generate FT-G2341R, FT-L2284P, FT-C1264R, FT-A2234D, and untagged Tg plasmids (supplemental Table S1). Primary antibodies were acquired from commercial sources and used at the indicated dilutions in immunoblotting buffer (5% bovine serum albumin in Tris-buffered saline pH 7.5, 0.1% Tween-20, and 0.1% sodium azide). Mouse monoclonal antibodies were used for the detection of KDEL (1:1000, Enzo Life Sciences, ADI-SPA-827), M2 anti-FLAG (1:1000, Sigma Aldrich, F1804). Polyclonal rabbit antibodies were used to detect Calnexin (1:1000, GeneTex, GTX109669), protein disulfide isomerase family A member 4 (PDIA4) (1:1000, Proteintech, 14712-1-AP), DNAJC10 (1:500, Proteintech, 13101-1-AP), thyroglobulin (1:1000, Proteintech, 21714-1-AP), UGGT1 (1:1000, Proteintech, 14170-1-AP), STT3A (1:2000, Proteintech, 12034-1-AP), and STT3B (1:2000, Proteintech, 15323-1-AP). Secondary antibodies were obtained from commercial sources and used at the indicated dilutions in 5% milk in Tris-buffered saline pH 7.5, 0.1% Tween-20 (TBS-T): Goat anti-mouse Starbright700 (1:10,000, Bio-Rad,12004158), Goat anti-rabbit IRDye800 (1:10,000, LI-COR, 926-32211), and Goat anti-rabbit Starbright520 (1:10,000, Bio-Rad,12005869).

### Cell Culture and Transfections

HEK293^DAX^ cells (32), HEK293T, and STT3A or STT3A KO HEK293T cells (33) were grown in Dulbecco’s modified Eagle’s medium (DMEM) supplemented with 10% fetal bovine serum (FBS), 1% L-glutamine (200 mM), and 1% penicillin (10,000U)/streptomycin (10,000 μg/ml). Cells were transiently transfected with respective FT Tg expression plasmids using a calcium phosphate method.

### Affinity Purification and MS Sample Preparation

A fully confluent 10 cm tissue culture plate (approximately 10^7^ cells) was used per condition. Cells were harvested by washing with PBS and incubating with 1 mM EDTA in PBS on ice. A cell scraper was then used to dislodge cells. Cells were harvested, washed once with PBS, and treated with 0.5 mM DSP (Thermo Scientific, PG82081) in PBS for 30 min at room temperature while rotating. Crosslinking was quenched by addition of 100 mM Tris pH 7.5 for 15 min. Lysates were prepared by lysing in RIPA buffer (50 mM Tris pH 7.5, 150 mM NaCl, 0.1% SDS, 1% Triton X-100, 0.5% deoxycholate and protease inhibitor cocktail (Roche, 4693159001), and protein concentration was normalized. Cell lysates were then precleared on 4B sepharose beads (Sigma, 4B200) at 4 °C for 1 h while rocking. Precleared lysates were immunoprecipitated with M2 anti-flag agarose resin (Sigma, A2220) or G1 Anti-DYKDDDDK affinity resin (GenScript, L00432) overnight at 4 °C while rocking. Resin was washed four times with RIPA buffer, and proteins were eluted twice in 75uL elution buffer (2% SDS, 1 mM EDTA, in PBS) by heating at 95 °C for 5 min. Eluted samples were precipitated in methanol/chloroform, washed three times with methanol, and air dried. Protein pellets were then resuspended in 3 μL 1% Rapigest SF Surfactant (Waters, 186002122) followed by the addition of 10 μL of 50 mM HEPES, pH 8.0 and 32.5 μL of H_2_O. Samples were reduced with 5 mM tris(2-carboxyethyl)phosphine (Sigma, 75259) at room temperature for 30 min and alkylated with 10 mM iodoacetimide (Sigma, I6125) in the dark at room temperature for 30 min. 0.5 μg of Trypsin (Promega, V511A or Thermo Scientific, PI90057) was then added and incubated for 16 to 18 h at 37 °C, shaking at 700 rpm. Peptides were reacted with TMT sixplex reagents (Thermo Fisher, 90066) in 40% v/v acetonitrile and incubated for 1 h at room temperature. Reactions were quenched by the addition of ammonium bicarbonate (0.4% w/v final concentration) and incubated for 1 h at room temperature. TMT-labeled samples for a given experiment were then pooled and acidified with 5% formic acid (Fisher, A117, v/v). Samples were concentrated using a speedvac and resuspended in buffer A (95% water, 4.9% acetonitrile, and 0.1% formic acid, v/v/v). Cleaved Rapigest SF surfactant was removed by centrifugation for 30 min at 21,100*g*.

### Liquid Chromatography—Tandem Mass Spectrometry

MudPIT microcolumns were prepared as previously described (34). Peptide samples were directly loaded onto the columns using a high-pressure chamber. Samples were then desalted for 30 min with buffer A (95% water, 4.9% acetonitrile, 0.1% formic acid v/v/v). LC-MS/MS analysis was performed using a Q-Exactive HF (Thermo Fisher) or Exploris480 (Thermo Fisher) mass spectrometer equipped with an Ultimate3000 RSLCnano system (Thermo Fisher). MudPIT experiments were performed with 10 μl sequential injections of 0, 10, 30, 60, and 100% buffer C (500 mM ammonium acetate in buffer A), followed by a final injection of 90% buffer C with 10% buffer B (99.9% acetonitrile, 0.1% formic acid v/v) and each step followed by a 130 min gradient from 5% to 80% B with a flow rate of 300 nl/min when using the Q-Exactive HF or 500 nl/min when using the Exploris480 on a 20 cm fused silica microcapillary column (ID 100 μm) ending with a laser-pulled tip filled with Aqua C18, 3 μm, 100 Å resin (Phenomenex). Electrospray ionization was performed directly from the analytical column by applying a voltage of 2.0 kV when using the Q-Exactive HF or 2.2 kV when using the Exploris480 with an inlet capillary temperature of 275 °C. Using the Q-Exactive HF, data-dependent acquisition of mass spectra was carried out by performing a full scan from 300 to 1800 m/z with a resolution of 60,000. The top 15 most abundant ions from each full scan were fragmented by higher-energy collisional dissociation using normalized collision energy of 38, 0.7 m/z isolation window, 120 ms maximum injection time, at a resolution of 15,000 scanned from 100 to 1800 m/z, and dynamic exclusion set to 60s. Using the Exploris480, data-dependent acquisition of mass spectra was carried out by performing a full scan from 400 to 1600 m/z at a resolution of 120,000. Top-speed data acquisition was used for acquiring MS/MS spectra using a cycle time of 3 s, with a normalized collision energy of 36, 0.4 m/z isolation window, 120 ms maximum injection time, at a resolution of 30,000 with the first mass (m/z) starting at 110. Peptide identification and TMT-based protein quantification was carried out using Proteome Discoverer 2.3 or 2.4. MS/MS spectra were extracted from Thermo Xcalibur.raw file format and searched using SEQUEST against a Uniprot human proteome database (released 03/2014 and containing 20,337 entries). The database was curated to remove redundant protein and splice-isoforms and supplemented with common biological MS contaminants. Searches were carried out using a decoy database of reversed peptide sequences and the following parameters: 10 ppm peptide precursor tolerance, 0.02 Da fragment mass tolerance, minimum peptide length of 6 amino acids, trypsin cleavage with a maximum of two missed cleavages, dynamic methionine modification of 15.995 Da (oxidation), static cysteine modification of 57.0215 Da (carbamidomethylation), and static N-terminal and lysine modifications of 229.1629 Da (TMT sixplex).

### Experimental Design and Statistical Rationale

A total of 13 6plex TMT LC-MS/MS batches were analyzed for this study (supplemental Table S2). The biological replicate co-immunoprecipitation samples were arranged in such a way that each Tg variant was directly paired against one another a minimum four times, with each batch including at least one mock IP control. In total, the analysis included 15 biological replicates for control and WT Tg samples, 12 biological replicates for L2284P and G2341R Tg samples, and 6 biological replicates for A2234D Tg samples. SEQUEST search results were filtered using Percolator to minimize the peptide false discovery rate to 1% and a minimum of two peptides per protein identification. TMT reporter ion intensities were quantified using the Reporter Ion Quantification processing node in Proteome Discoverer 2.3 or 2.4 and summed for peptides belonging to the same protein, including razor peptides.

To identify true interactors from nonspecific background, TMT intensities first underwent a log_2_ transformation and were then median normalized using the formula: $I_{n,\;TMT\alpha }^{norm}=\;I_{n,\;TMT\alpha }^{unnorm}•\frac{\sum_{TMT_{\gamma }}^{TMT_{\alpha }}M}{M_{TMT\alpha }}$. Here, $I_{n,\;TMT\alpha }^{norm}$ and $I_{n,\;TMT\alpha }^{unnorm}$ are the unnormalized and normalized TMT intensities for a given protein *n* found in TMT channels α-γ, and M is the median TMT intensity value for TMT channels α-γ. TMT ratios were then calculated between respective Tg AP and control TMT channels using formula: ${\log\;}_{2}\;I_{n,\;TMT\alpha }^{norm}\;-\;{\log\;}_{2}\;I_{n,\;TMT\gamma }^{norm}$ (supplemental Fig. S1, *A–B*, supplemental Table S3). The mean of log_2_ interaction differences was then calculated across the multiple LC-MS batches consisting of 15 biological replicates for control and WT Tg samples, 12 biological replicates for L2284P and G2341R Tg samples, and 6 biological replicates for A2234D Tg samples. Missing protein identification and TMT quantifications were ignored. Significance of interaction differences was calculated using a paired, parametric, two-tailed *t* test of log2 $I_{n,\;TMT\alpha }^{norm}$. Only proteins quantified in at least two biological replicate co-immunoprecipitation conditions were carried forward. The *p* values were adjusted to respective q values using the method described by Storey *et al*. (35) for multiple testing correction to estimate false discovery rate, where the q value of a feature describes the proportion of false positives among all features equally or more extreme than the one observed. A previously described method was used to designate true interactors from nonspecific background (36). In short, the function *y* = *c*/(*x* − *x*_0_) was used, where c = curvature and *x*_0_ = minimum fold change. *x*_0_ was set as one standard deviation, rounded to a single decimal place, of the average log_2_ protein abundance differences of the respective TMT channels containing Tg variants compared with the respective TMT channels containing the mock AP controls. The c parameter was optimized to separate true interactors from false positives, focusing on proteins found to be implicated within the secretory pathway using GO pathway enrichment analysis (supplemental Fig. S1, *D–H* and supplemental Table S3). Tg interactors were identified for WT and mutant Tg constructs individually. A cumulative list of identified interactors was then used for WT *versus* mutant Tg comparisons (supplemental Table S4).

To compare WT *versus* mutant Tg high-confidence interactors, TMT intensities were first normalized using formula: $I_{n,\;TMT\alpha }^{norm}=\;I_{n,\;TMT\alpha }^{unnorm}•\frac{\sum_{TMT_{\gamma }}^{TMT_{\alpha }}I_{Tg}^{unnorm}}{I_{Tg,\;TMT\alpha }^{unnorm}}$. Here, $I_{n,\;TMT\alpha }^{norm}$ and $I_{n,\;TMT\alpha }^{unnorm}$ are the unnormalized and normalized TMT intensities for a given protein *n* found in TMT channels α-γ, and $I_{Tg}^{unnorm}$ is the unnormalized TMT intensity value for Tg in a given TMT channel α-γ. Significance of interaction differences was calculated using a paired, parametric, two-tailed *t* test of log_2_ $I_{n,\;TMT\alpha }^{norm}$. The *p* values were adjusted to respective q values using the method described by Storey *et al.* (35) for multiple testing correction to estimate false discovery rate, where the q value of a feature describes the proportion of false positives among all features equally or more extreme than the one observed.

For pathway enrichment analysis of identified proteins, EnrichR was used, and GO Cellular Component 2018 terms were used to differentiate secretory pathway-associated proteins from background (supplemental Table S5) (37). Tg interactors were similarly analyzed using GO Molecular Function 2018 terms. The raw dataset used for the mass spectrometry interactome characterization experiments showing protein identification, coverage, and quantification is included in supplemental Table S6. Peptide identification information is contained in supplemental Table S7. Spectrum and result files are available via ProteomeXchange under identifier PXD019427.

### Immunoblotting, SDS-PAGE, and Immunoprecipitation

Cell lysates were prepared by lysing in RIPA buffer with protease inhibitor cocktail, and protein concentrations were normalized. Lysates were then denatured with 1X Laemmli buffer +100 mM DTT and heated at 95 °C for 5 min before being separated by SDS-PAGE. Samples were transferred onto polyvinylidene difluoride membranes (Millipore) for immunoblotting and blocked in TBS-T. Primary antibodies were incubated either at room temperature for 2 h or overnight at 4 °C. Membranes were then washed four times with TBS-T and incubated with secondary antibody in 5% nonfat dry milk/TBS-T either at room temperature for 1 h or overnight at 4 °C. Membranes were washed four times with TBS-T and then imaged using a ChemiDoc MP Imaging System (BioRad). Quantification was performed using Image Lab Software (BioRad). For Tg immunoprecipitation, normalized lysates were incubated with M2 anti-flag agarose resin or G1 Anti-DYKDDDDK affinity resin overnight at 4 °C. Resin was then washed four times with RIPA buffer, and samples were eluted using 3X Laemmli buffer with 100 mM DTT. For immunoblot confirmation of Tg interactors, samples were processed exactly as described above for interactome characterization via affinity purification–mass spectrometry, including addition of 0.5 mM DSP cross-linker. Proteins were eluted once with elution buffer (2% SDS, 1 mM EDTA, in PBS) by heating at 95 °C for 5 min.

### Cycloheximide Chase Assay

Cells transfected with Tg variants were plated onto poly-D-lysine coated wells in 6-well dishes. Cells were washed twice with 2 ml of media treated with CHX (50 μg/ml), then chased with 1 ml of CHX-treated media and collected at various time points. Cells were harvested by aspirating media, washing cells twice with 2 ml of cold PBS, and lysing in 1 ml RIPA buffer with protease inhibitor cocktail (Roche, 4693159001). Collected media was spun down at 400*g* for 5 min to pellet any floating cells. Cell lysate and media was subjected to immunoprecipitation with M2 anti-flag agarose resin or G1 Anti-DYKDDDDK affinity resin overnight at 4 °C. Resin was then washed four times with RIPA buffer, and samples were eluted using 3X Laemmli buffer with 100 mM DTT. Eluted samples were separated by SDS-PAGE and transferred to polyvinylidene difluoride membrane and probed with primary and secondary antibody as described above.

### ^35^S Pulse Chase Assay

Cells transfected with Tg variants were plated onto poly-D-lysine coated wells in 6-well dishes. Cells were incubated with methionine and cysteine depleted DMEM supplemented with glutamine, penicillin/streptomycin, and 10% FBS at 37 °C for 30 min. Cells were then metabolically labeled in DMEM depleted of methionine and cysteine and supplemented with EasyTag ^35^S Protein Labeling Mix (Perkin Elmer, NEG772007MC), glutamine, penicillin/streptomycin, and 10% FBS at 37 °C for 30 min. Afterward, cells were washed twice with DMEM containing 10 X methionine and cysteine, followed by a burn off period of 30 min in normal DMEM. Cells were then chased for the respective time period with normal DMEM, lysed with 500uL of RIPA buffer with protease inhibitor cocktail and 10 mM DTT. Cell lysates were diluted with 500uL of RIPA buffer with protease inhibitor cocktail and subjected to immunoprecipitation with G1 anti-DYKDDDDK affinity resin overnight at 4° C. After three washes with RIPA buffer, protein samples were eluted with 3× Laemmli buffer with 100 mM DTT heating at 95 °C for 5 min. Eluted samples were then separated by SDS-PAGE, and gels were dried and exposed on a storage phosphor screen. Radioactive band intensity was then measured using a Typhoon Trio Imager (GE Healthcare) and quantified by densitometry in Image Lab (BioRad).

### EndoH and PNGaseF Treatment

Cells were lysed in either RIPA or TNI (50 mM Tris pH 7.5, 250 mM NaCl, 1 mM EDTA, and 0.5% IGEPAL CA-630) buffer with protease inhibitor cocktail, denatured, and digested with EndoH or PNGase F per the manufacturer specifications (New England BioLabs).

## Results

### Distinct Thyroglobulin Mutants Present Common Secretion Defects

Tg is a large 330 kDa multidomain protein consisting of extensive cysteine-rich repeat regions and a C-terminal cholinesterase-like domain (ChEL) (Fig. 1*A*, supplemental Fig. S2, *A–B*) (38). We focused on a set of single-point mutations that lead to impaired Tg secretion in human CH patients (A2234D and C1264R) and in a mouse model of thyroid hormone deficiency and goiter (L2284P) (17, 39, 40). A2234D and L2284P occur in the ChEL domain, which serves as an intramolecular chaperone playing a critical role in Tg folding, dimerization, and secretion (41, 42). Our analysis also included a previously uncharacterized ChEL mutation at a conserved glycine (G2341R), which is located adjacent to L2284 and A2234. We contrasted the ChEL mutations to the C1264R variant in the hinge/flap region (Fig. 1*A*, supplemental Fig. S2, *A–D*, supplemental Table S8).

Thyroid hormone production is initiated by the biogenesis and secretion of Tg, which is subsequently iodinated and stored in the thyroid follicular lumen. Once thyrocytes are stimulated, Tg is endocytosed and degraded in the lysosome, leading to the liberation of T3 and T4 hormones (Fig. 1*B*). We transiently transfected HEK293T cells with FLAG-tagged expression constructs of either WT or the respective mutant Tg variants. Overexpression of these constructs through transient transfection mimics that of high levels of endogenous Tg expression in thyroid tissue. We detected all Tg variants at similar levels in lysate samples, while only WT Tg was detected in the media, confirming the secretion defect of CH mutations (Fig. 1*C*). C1264R Tg was occasionally detected at trace amounts in the media indicating low residual secretion (<1–2% of WT). These results are in accordance with previous Tg studies (17, 39, 43, 44). WT Tg undergoes extensive glycosylation within the ER before being trafficked and further modified in the Golgi apparatus, while the folding-incompetent CH-associated mutations are trapped within the ER preventing Golgi modifications. To investigate Tg localization and glycosylation state, we performed EndoH digestions on the transfected HEK293T lysates. All mutants were EndoH sensitive, indicating they had not traversed the medial Golgi apparatus as EndoH specifically cleaves ER-associated high-mannose glycans. In contrast, WT Tg separated into two distinct EndoH-resistant and EndoH-sensitive populations indicating that WT Tg was able to fold within the ER and traverse the secretory pathway (Fig. 1, *D* and *E*). The low level of intracellular EndoH-resistant WT Tg indicates that this proteoform gets rapidly secreted. Overall, our results confirm that FLAG epitope–tagged Tg constructs do not show altered processing and serve as a useful model system to probe PQC dynamics for WT and CH-associated, secretion-deficient Tg mutants.

### Defining the Tg Proteostasis Interactome

**Fig. 2:**
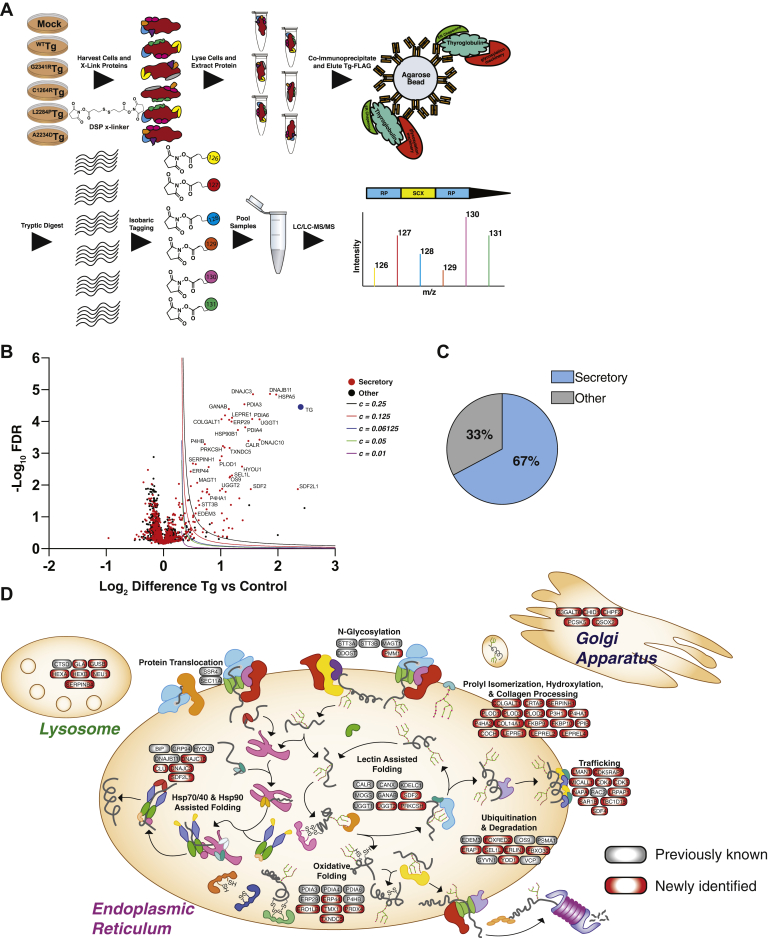
**Defining the Tg interactome using multiplexed quantitative AP-MS.***A*, schematic detailing the multiplexed quantitative interactomics workflow utilizing *in situ* crosslinking, affinity purification—mass spectrometry (AP-MS), and tandem mass tags (TMT) for relative quantification of identified interactors to delineate interaction changes from WT to mutant variants. *B*, volcano plot showing TMT enrichment ratios (log_2_ difference all Tg channels *versus* all mock channels) *versus* −log10 adjusted *p* values (Storey q values) for coimmunoprecipitated proteins (n = 13 biological replicates). Variable cutoffs were used to optimize confident interactors of Tg. Optimization described in supplemental Figure S1 and supplemental Table S3. *C*, proteins found to be confident interactors with Tg are enriched within the secretory pathway. *D*, Schematic detailing newly identified Tg interactors (*red*) compared with previously publishes interactors (*gray*). Tg interactors are organized by biological function and organellar localization. Tg, thyroglobulin.

To identify protein–protein interactions implicated in the aberrant processing of CH-associated mutations, we implemented an affinity purification–mass spectrometry method coupled with tandem mass tag (TMT) labeling to allow for multiplexed identification and quantification of interacting proteins (Fig. 2*A*) (45). Transient interactions between Tg and PN components were captured using the cell permeable and reversable cross-linker dithiobis(succinimidyl propionate) (DSP) (46). DSP covalently links interacting proteins before immunoprecipitation, whereas the disulfide linker within DSP allows the protein partners to be efficiently dissociated under reducing conditions once immunoprecipitation is complete (Fig. 2*A*). We optimized cross-linking and stringent wash conditions during the affinity purification to capture Tg interactions with known PN components. Interactions with GRP94 and HYOU1 were only observable with addition of DSP, whereas BiP and PDIA4 were much more strongly detectable with DSP (supplemental Fig. S1*I*). We first sought to define the interactomes for WT Tg and each of the mutant variants (G2341R, L2284P, A2234D, and C1264R). We employed a mock AP using transfection of an untagged WT Tg control construct or fluorescent proteins (enhanced green fluorescent protein or tdTomato fluorescent protein) to delineate high confidence interactors from background proteins (Fig. 2*A*). We further optimized normalization methods and cutoffs to confidently identify interactors pertaining to protein folding, trafficking, and secretion likely to play a role in Tg processing (supplemental Fig. S1, *A–B* and supplemental Table S3) (36, 37). Enrichment of interaction partners was comparable when fluorescent proteins or untagged Tg was used as mock AP controls (supplemental Fig. S1*C*) enabling us to combine all batches. We then identified interacting proteins for each individual Tg construct (supplemental Figs. S1*D–H*, supplemental Table S4) and defined a cumulative list of 186 confidently identified interactors across all Tg constructs (Fig. 2*B*, supplemental Table S4). We defined this list as the Tg interactome and focused on these proteins for subsequent analyses. Using gene ontology (GO) enrichment analysis, 67% of the Tg interactome was enriched in components belonging to PQC within the secretory pathway (Fig. 2*C* and supplemental Table S5), including heat shock protein 70 kDa and 40 kDa chaperone/co-chaperone system (Hsp70/40) and heat shock protein 90 (Hsp90) chaperones and co-chaperones, N-glycosylation machinery, glycoprotein folding lectins, components involved in disulfide bond formation, ERAD, as well as lysosomal and Golgi localized proteins (37). We confirmed that the majority of secretory PQC factors were identified and quantified consistently across biological replicate mass spectrometry batches (median identified in 62% of runs, supplemental Fig. S1*J*).

Our dataset confirms previously known binding partners, such as GRP94, BiP, PDIA3, CANX, and CALR (14, 47), but it greatly expands the limited list of previous interactors (Fig. 2*D*). When comparing Tg interactors identified here to other protein–protein interaction databases such as Bioplex, BioGRID, or STRING, our dataset is far more rich in interactions with relevant proteostasis components (supplemental Table S9). Tg is absent from the Bioplex database, and BioGRID only contains three interactors of Tg, BiP, P4HB, and Tg itself as it forms a homodimer. BiP and P4HB are both found within our dataset. STRING contains five experimentally determined interactors of Tg, although none of these were proteostasis components. Overall, the comparisons highlight that our interactomics dataset provides unprecedented insights into PN interactions associated with Tg processing.

We identified additional ER Hsp40 co-chaperones, including DnaJ heat shock protein family member C3, DnaJ heat shock protein family member B11 (DNAJB11), and DnaJ heat shock protein family member C10 (DNAJC10), as well as the nucleotide exchange factor HYOU1, that can bind Tg and coordinate with the ER Hsp70 chaperone BiP to influence quality control decisions (30, 48). Generally, DNAJB11 and BiP are known to be “pro-folding” proteostasis components as they are able to generally bind sites dispersed throughout the sequence of ER clients. Conversely, DNAJC10 and HYOU1 have been shown to distinctly recognize rare sequences, with high aggregation propensity within ER client (30). DnaJ heat shock protein family member C3 is also thought to broadly bind substrate domains dispersed throughout the client sequence but have not been characterized as well as its DNAJB11 and DNAJC10 counterparts (30, 49) The enrichment in disulfide bond processing components is consistent with a strong dependence on oxidative folding pathways with Tg containing 122 cysteine residues and 61 disulfide bonds (38). Protein disulfide isomerase family A member 1/P4HB, PDIA3/ERp57, PDIA4/ER protein 72, protein disulfide isomerase family A member 6 (PDIA6)/ER protein 5, and protein disulfide isomerase family A member 9/ER protein 29 were previously known to associate with Tg (14, 15, 50), but we additionally identified protein disulfide isomerase family A member 10/ER protein 44, thioredoxin domain containing 5/ER protein 46, and thioredoxin-related transmembrane protein 1, among others (Fig. 2*D*). These redox enzymes form intricate networks within the ER, playing key roles in protein folding and degradation (51). ERAD factors (OS9 endoplasmic reticulum lectin [OS-9], ER degradation enhancing alpha-mannosidase–like [EDEM] 3, SEL1L adaptor subunit of ERAD E3 ubiquitin ligase [SEL1L]), which have been presumed to interact with Tg but not confirmed (4), along with new factors such as RAD-dependent oxidoreductase domain containing 2 (FOXRED2) were also identified (Fig. 2*D*). OS-9 and SEL1L are key components of the HRD1-SEL1L ubiquitin ligase complex responsible for the ubiquitination of many ERAD substrates, whereas EDEM3 and FOXRED2 act more upstream during the ERAD process (52, 53, 54, 55). Furthermore, we detected previously known and novel interactors involved in glycoprotein folding and processing such as glucosidase II alpha subunit (GANAB), lectin mannose binding 1, UDP-glucose glycoprotein glucosyltansferase 1 (UGGT1), as well as other lectins and glycan modifying enzymes.

When analyzing construct-specific interactions (supplemental Fig. S3), we observed that WT Tg interacted with RAC2, TUBA4A, and PLEC, which play a role in post-ER protein trafficking and secretion. Interestingly, we noticed that mutations localized to the ChEL region exhibited construct-specific interactions with a different set of vesicle trafficking components. The GTPase activator TBC1D15 interacted with G2341R Tg specifically, while the small GTPase SAR1B bound to L2284P Tg. A2234D Tg showed specific interactions with ARHGAP21, another GTPase activator, and TFG, a required component for secretory cargo trafficking from the ER to the Golgi apparatus. Because these mutations are not secreted, these interactions may suggest CH-associated mutations can be sequestered to ER exit sights and get retained in the secretory pathway. Construct specific interactions for C1264R Tg showed broader biological function. For example, the ER membrane complex subunit EMC1, the ER-localized peroxidase PXDN, the ER lipid raft–associated protein ERLIN1, and glycan-modifying enzyme B3GALT6 were all found to interact with C1264R. Overall, our analysis validated 27 previously identified Tg interactors and described 159 new PN interactions (Fig. 2*D* and supplemental Table S4).

### The Secretion Defect of Tg Mutants Is Associated With Common Increases in Proteostasis Interactions

**Fig. 3:**
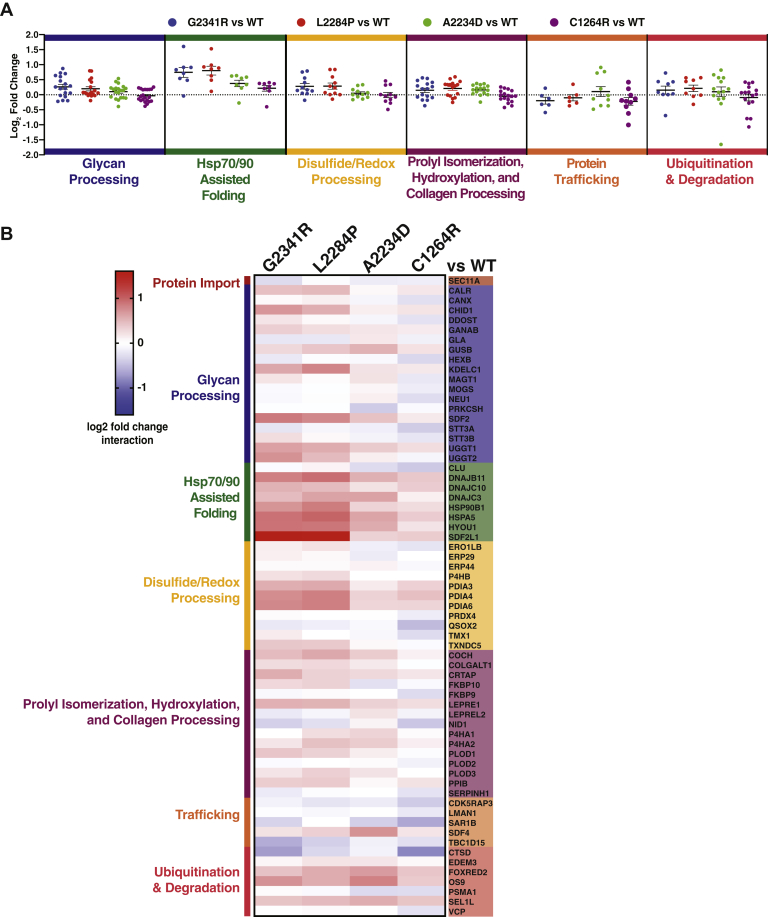
**The secretion defect of Tg mutants is associated with both common and mutant specific changes in proteostasis interactions.***A*, dot plots displaying aggregate interactome changes of proteostasis pathways between the different mutant Tg variants (G2341R, L2284P, A2234D, C1264R) compared with WT. Proteostasis factors are grouped based on biological function as in Figure 3*B,* and dots represent interaction changes for individual high-confidence interactors of Tg belonging to each group. Source data found in supplemental Table S10. *B*, heatmap displaying altered interactions of mutant Tg variants with individual proteostasis components that were identified as high-confidence interactors. Interactors are grouped by biological function as in Figure 3*A*. Source data found in supplemental Table S10. Tg, thyroglobulin.

Next, we quantified interaction fold changes for the specific mutants relative to WT Tg to determine what factors may govern the aberrant PQC processing and secretion defects (Fig. 3, *A*–*B*, supplemental Fig. S4*A*, supplemental Tables S10 and S11). To account for small difference in Tg abundances, we normalized the TMT intensities of high-confidence interactors to the Tg intensities (supplemental Fig. S4*B*). Remarkably, when comparing CH-associated mutant interactomes with that of WT, many of the quantified interaction changes were similar across all CH-associated mutants. This held true particularly for factors involved in Hsp70/40- and Hsp90-assisted protein folding, including the Hsp70 and Hsp90 chaperones BiP and GRP94 along with co-chaperones DNAJB11 and DNAJC10. We observed similar increases with disulfide/redox-processing enzymes such as protein disulfide isomerases PDIA3, PDIA4, and PDIA6. We validated increased interactions between mutant Tg variants and GRP94, BiP, PDIA4, PDIA6, and DNAJC10 by Co-AP followed by quantitative Western blot (supplemental Fig. S4, *C–E*). Importantly, these increased interactions were not the results of changes in protein levels of these PN components in response to Tg overexpression. We confirmed that protein levels of several PN components controlled by the unfolded protein response remain unchanged (supplemental Fig. S4*D*).

**Fig. 4:**
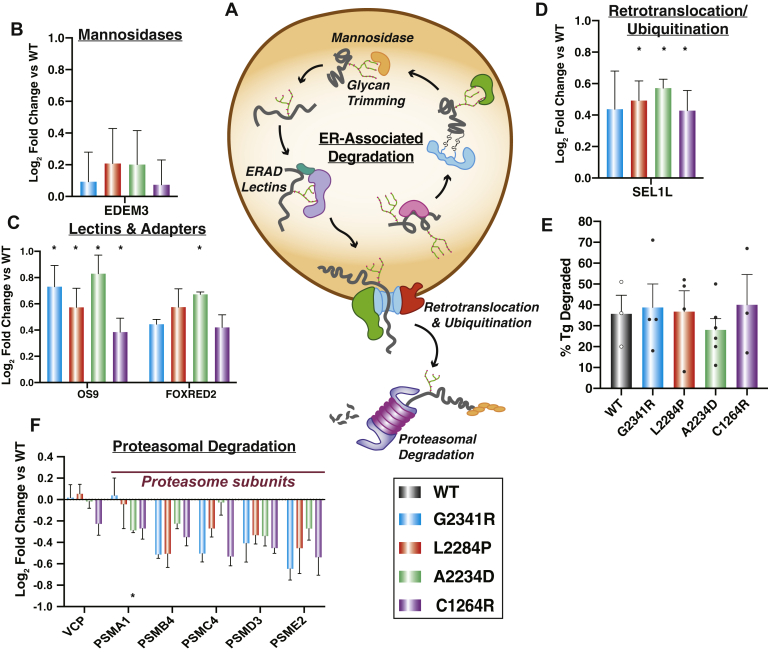
**Tg mutants are increasingly routed toward ER-associated degradation machineries but not degraded at faster rates.***A*, schematic detailing the ERAD pathway: degradation factors targeting proteins through glycan trimming, subsequent retrotranslocation and ubiquitination, followed by proteasome-mediated degradation in the cytosol. *B*–*D*, interaction changes of Tg mutants compared with WT with individual ERAD factors that were identified as high-confidence interactors of Tg. Error bars show SEM. Student’s parametric *t* test with adjusted *p* values (Storey q values) was used to determine significant changes. ∗q < 0.05. *B*, Mannosidases responsible for glycan trimming. *C*, an ERAD-specific lectin (OS-9) and another ERAD factor (FOXRED2), *D*, a subunit of the retrotranslocation complex. *E*, plot showing the percentage of Tg degradation measured in HEK293^DAX^ cells 4 h after treatment with 50 μg/ml of cycloheximide to block new protein translation. Error bars show SEM for three to six biological replicates. There is no significant difference in degradation for any of the Tg mutants compared with WT. Student’s parametric *t* test was used to determine significant changes (*p* < 0.05). G2341R: *p* = 0.849, L2284P: *p* = 0.942, A2234D: *p* = 0.464, C1264R: *p* = 0.813. Representative Western blots for the CHX chase experiments are shown in supplemental Figure S5B. *F*, interaction changes of Tg mutants compared with WT for cytosolic proteins involved in proteasome-mediated protein degradation. Error bars shows SEM. Student’s parametric *t* test with multiple testing correction (Storey q values) was used to determine significant changes. ∗q < 0.05. CHX, a cycloheximide; ER, endoplasmic reticulum; ERAD, ER-associated degradation; Tg, thyroglobulin.

Additionally, the enzymes responsible for marking and directing ER clients for ERAD including EDEM3, FOXRED2, OS9, and SEL1L all showed consistently increased interactions with Tg mutants compared with WT (Fig. 4, *A*–*D*) (52, 53, 54, 56). This observation prompted us to test whether the CH-associated mutant Tg variants are degraded at a higher rate. To monitor potential changes in degradation rates of the Tg constructs, we employed a cycloheximide (CHX) chase assay (supplemental Fig. S5*A*). Approximately one-third of WT Tg was secreted after 4 h (supplemental Fig. S5*B*). As previously noted, none of the CH-associated Tg mutants were secreted. When monitoring Tg degradation, all constructs showed similar rates of observed degradation on the scale of 30 to 40% after 4 h of CHX treatment (Fig. 4*E* and supplemental Fig. S5*B*). To ensure that the loss of Tg secretion can be attributed to degradation and does not correspond to aggregated protein, we quantified WT and mutant Tg in the insoluble cell pellet. While a fraction of Tg could be found in the pellet, consistent with prior observation (57, 58), the degree of aggregation remained similar for all variants (supplemental Fig. S5, *C–D*). Overall, the degradation rates are consistent with prior studies (59) and indicate that despite increased engagement with ERAD targeting factors, mutant Tg degradation rates are unaffected.

To further investigate why degradation rates remain unchanged for the mutant variants, we next looked at downstream proteostasis factors involved in ERAD after retrotranslocation of proteins into the cytosol. We detected Tg interactions with valosin containing protein (VCP/p97), the ATPase involved in extracting substrates from the ER, as well as several subunits of the proteasome (Fig. 4, *A* and *F*). Interactions between Tg mutant variants and VCP were mostly unchanged relative to WT, whereas proteasome subunit interactions were consistently decreased for all mutants. This reduced engagement of CH-associated Tg mutants with the degradation machinery may provide an explanation why these variants are not degraded to a larger extend despite their increased targeting to glycoprotein ERAD relative to WT.

Overall, the interactomics data suggest that CH-associated mutant Tg displays common PQC defects linked to prolonged chaperoning facilitated largely by Hsp70/40, Hsp90, and disulfide/redox folding pathways, as well as increased associations with ER luminal ERAD components. Interestingly, in many cases interaction fold changes were slightly higher for all ChEL domain Tg mutants, G2341R, L2284P, and A2234D than for the C1264R mutant occurring in the hinge/flap region (Fig. 3*B*, supplemental Table S10). This parallels our data and previous secretion data exhibiting residual C1264R-Tg secretion (44) (Fig. 1*C*) and suggests that the Tg PQC defects are more profound when mutations occur in the ChEL domain.

### CH-Associated Tg Exhibits Mutation-Specific Changes in Engagement With the ER-Lectin Chaperone Network

While identifying common changes in PN interactions across CH-associated mutants provides new insights on conserved Tg processing mechanisms, we wanted to further explore the data to investigate whether any mutation-specific PN interaction changes occurred that may define unique PQC defects associated with each mutation. In the case of disulfide/redox processing, mutants exhibited distinct dependencies on PN components, which may play unique roles in processing the defective proteins. C1264R uniquely exhibited significantly decreased interactions with the thioredoxin-related transmembrane protein 1, A2234D had decreased interactions with ER protein 29, and conversely L2284P showed significantly increased interactions with P4HB (Fig. 3*B*).

**Fig. 5:**
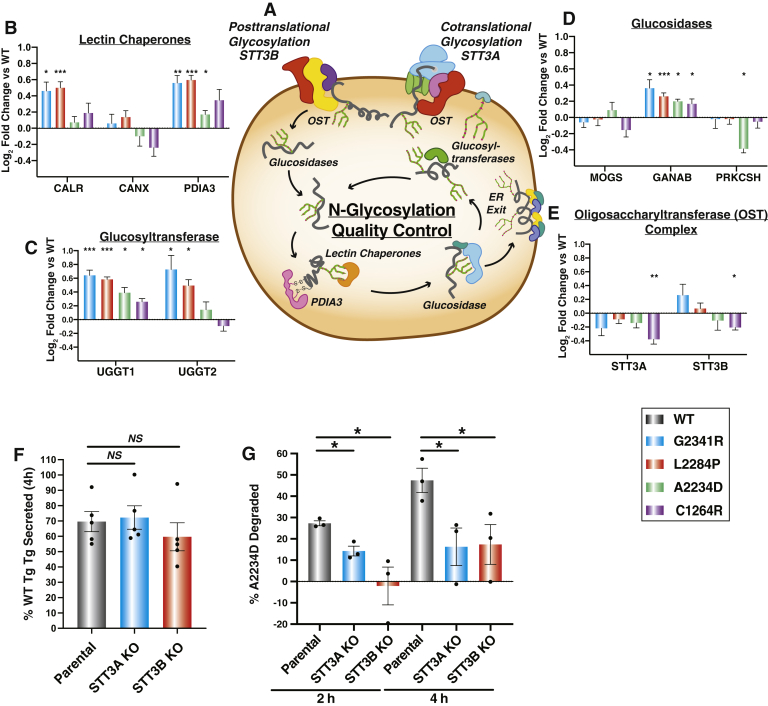
**Perturbation of N-linked glycosylation distinctly impacts A2234D Tg.***A*, schematic detailing the N-glycosylation and lectin-mediated folding pathway. Glycosylation is carried out by two distinct OST complexes containing STT3A or STT3B as catalytic subunits. Glucosidases then trim terminal glucose residues, lectin chaperones (CANX, CALR), and PDIA3 assist in folding, followed by further glucose trimming. Subsequently, glucosyltransferases (UGGT1, UGGT2) serve as quality control sensors to re-glucosylate improperly folded proteins for iterative chaperoning cycles. *B–E*, interaction changes of Tg mutants compared with WT with individual N-glycosylation quality control factors that were identified as high-confidence interactors of Tg. Error bars show SEM. Student’s parametric *t* test with adjusted *p* values (Storey q values) was used to determine significant changes. ∗q < 0.05, ∗∗q < 0.005, ∗∗∗q < 0.0005. *B*, lectin chaperones and lectin-associated protein disulfide isomerase PDIA3. *C*, glucosyltransferases sensing misfolded proteins. *D*, glucosidases involved in glycan trimming. *E*, catalytic subunits of the OST complex STT3A responsible for cotranslational glycosylation and STT3B responsible for posttranslational glycosylation. *F*, comparison of WT Tg secretion in parental, STT3A, or STT3B KO HEK293T cells. WT Tg was transiently transfected into the respective cells, and newly synthesized proteins were metabolically labeled for 30 min with ^35^S and then chased with unlabeled media. ^35^S-labeled protein was quantified in the media, and lysate after 4 h % Tg secreted was calculated as $Tg_{media,\;4h}/\left(Tg_{lysate,\;0h}+Tg_{media,\;0h}\right)$. Error bars represent SEM of three to four biological replicates. STT3A or STT3B KO do not significantly alter WT secretion. Student’s parametric *t* test was used to determine significant changes in Tg secretion. Representative autoradiograms are shown in supplemental Figure S5*G*. *G*, comparison of A2234D Tg degradation in parental, STT3A, or STT3B KO HEK293T cells. A2234D Tg was transiently transfected into the respective cells and subjected to the same ^35^S-pulse labeling scheme as in F. % Tg degraded was calculated as $1-\;\left(Tg_{lysate,\;4h}/Tg_{lysate,\;0h}\right)$. Error bars represent SEM of three to four biological replicates. Representative autoradiograms are shown in supplemental Figure S5*H*. Student’s parametric *t* test was used to determine significant (∗*p* < 0.05) changes in Tg degradation. OST, oligosaccharyl transferase complex; Tg, thyroglobulin; UGGT1, UDP-glucose glycoprotein glucosyltansferase 1;UGGT2, UDP-glucose glycoprotein glucosyltansferase 2.

The protein processing class where we noticed the most unique changes were those associated with glycan processing. We detected significant and distinct deviations across CH-associated Tg interactions with PN components involved in N-glycosylation, and the CANX/CALR lectin folding pathway (Fig. 5, *A*–*E*). Interactions with CALR, the soluble paralog of CANX, were significantly increased for G2341R and L2284P relative to WT Tg, but not for C1264R and A2234D. PDIA3, the protein disulfide isomerase family member known to specifically complex with either CANX or CALR, exhibited similarly increased interactions with G2341R and L2284P (60, 61) (Fig. 5*B*). Significantly increased interactions for all mutations were also observed for the glucosyltransferase UGGT1, as well as GANAB (Fig. 5, *C*–*D*). GANAB and UGGT1 act as the gate-keepers for glycoprotein folding. As GANAB sequentially cleaves the two innermost glucose residues of the ER-associated N-linked oligosaccharide precursor, regulating initial client entry and exit from the CANX/CALR lectin folding pathway, UGGT1 re-glycosylates misfolded ER glycoprotein clients, regulating their re-entry into the CANX/CALR lectin folding pathway (Fig. 5*A*) (61, 62). While the lectin folding pathway is of clear importance, all of these interactions occur downstream of the oligosaccharyl transferase (OST) complex which facilitates N-glycosylation of proteins (63). We hypothesized that the observed changes in ER lectin chaperone network engagement could stem from altered engagement with the OST complex. We noticed mutation-specific changes in engagement with the two different OST isoforms. In one isoform containing the catalytic oligosaccharyl transferase complex catalytic subunit A (STT3A) subunit, the OST is largely associated with the translocon channel and facilitates co-translational glycosylation of ER client proteins (64). The other OST isoform containing oligosaccharyl transferase complex catalytic subunit B (STT3B) is largely associated with posttranslational glycosylation of ER client proteins (64). Most CH-associated Tg mutants showed varying degrees of decreased interactions with the STT3A catalytic subunit relative to WT. In contrast, interaction changes with STT3B were divergent for mutant variants relative to WT Tg. G2341R and L2284P exhibited modestly increased or unchanged interactions, whereas A2234D and C1264R showed decreased interaction (Fig. 5*E*). We confirmed by Co-AP Western blot that A2234D and C1264R displayed decreased interactions with STT3B compared with WT and that these changes were distinct from G2341R and L2284P (supplemental Fig. S6*A*). Overall, our findings reveal mutation-specific PQC defects for the different CH-associated Tg mutants and their engagement and processing through the CANX/CALR lectin folding pathway. These changes may stem from subtle differences in engagement with the upstream OST complex, which mediates the entry into the CANX/CALR lectin folding pathway (Fig. 5*A*).

**Fig. 6:**
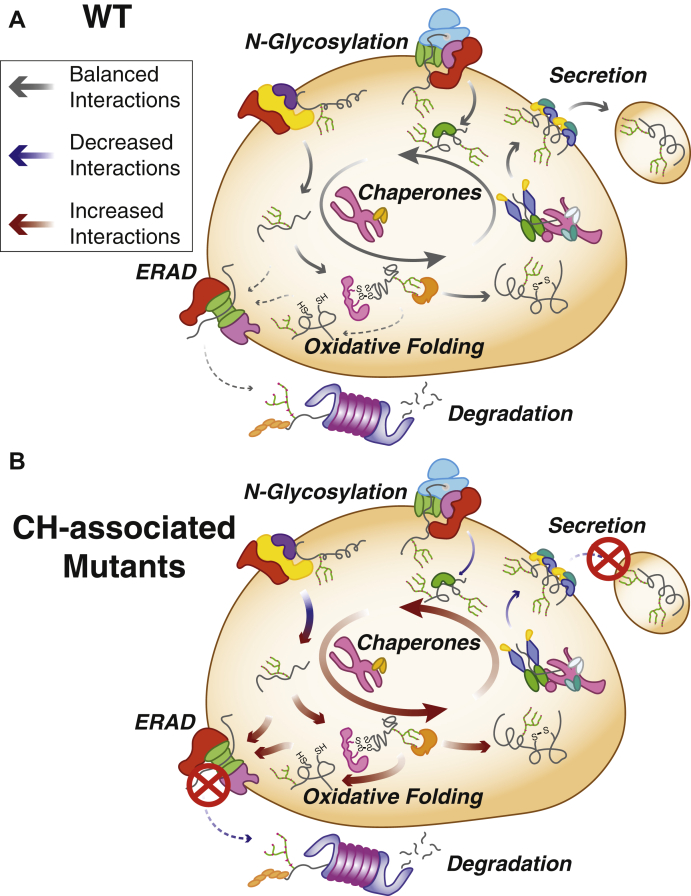
**Model for common and mutant-specific proteostasis interactome changes mediating the secretion defect of CH-associated Tg variants.***A*, in the case of WT, Tg processing (top) the delicate balance of proper chaperoning, posttranslational modification, and trafficking is maintained to provide sufficient Tg processing, secretion, and subsequent hormone production (indicated by the arrow size and color code denoted in the key). *B*, in the case of secretion-defective, CH-associated Tg mutants (bottom), this balance between chaperoning is shifted in such a way that increased chaperoning and engagement with oxidative folding enzymes, possibly stemming from altered engagement with the OST complex dominates Tg processing (indicated by the arrow size and color code denoted in the key). Additionally, mutant Tg is increasingly marked for degradation, yet inefficient retrotranslocation or decreased engagement by the proteasome leads to degradation rates remaining consistent compared to WT. CH, congenital hypothyroidism; OST, oligosaccharyl transferase complex; Tg, thyroglobulin.

### Perturbation of N-Linked Glycosylation Distinctly Impacts Tg Mutant

Tg contains 17 N-glycosylation sites and therefore depends heavily on the lectin-folding pathway, but little investigation has been dedicated to understanding how OST engagement, particularly in the case of CH-associated mutations, may affect downstream Tg processing (4). Therefore, we chose to focus on elucidating the role of the two different STT3A- and STT3B-dependent OST isoforms on downstream Tg processing. Individual glycosylation sites on protein clients display varying specificity toward glycosylation by either STT3A, STT3B, or both isoforms of the OST (33). Specific OST isoform dependencies for Tg N-glycosylation are not well studied, further motivating our investigation into the changes in OST engagement and downstream processing observed for CH-associated mutants. To assess the functional implications of these changes with OST engagement identified in our dataset, we monitored the effects of isoform-specific knockouts of STT3A or STT3B OST isoforms on Tg secretion and degradation (33, 64, 65). We transfected Tg variants into HEK293T STT3A^–/–^ and STT3B^–/–^ knockout cell lines and monitored protein levels in the media and the lysate under steady-state conditions. Knockout of either OST subunit did not abolish WT Tg secretion nor rescue secretion of any CH mutants (supplemental Fig. S6B).

Next, we followed Tg processing via CHX chase assay and ^35^S pulse-chase labeling to measure secretion and degradation rates (supplemental Figs. S5*A* and S6*C*). STT3A and STT3B knockout led to a small decrease in the WT Tg secretion rates when measured via CHX assay (supplemental Fig. S6, *D–E*). We then went on to quantify the degradation of CH-associated mutants (supplemental Fig. S6, *D* and *F*). Given the high variability in degradation measurements using the CHX chase assay, we employed the ^35^S pulse-chase labeling scheme to mitigate any complications resulting from inhibiting protein synthesis (supplemental Fig. S6, *G–H*). In particular, we focused on A2234D Tg as this mutation is proximal to two N-glycosylation sequons found in the ChEL domain known to act as an intramolecular chaperone (16, 42). We hypothesized that A2234D may alter the engagement of these sequons with the OST complex, altering Tg glycosylation and downstream processing. When measured using the ^35^S assay, STT3A and STT3B KO cells did not alter WT Tg secretion (Fig. 5*F*, supplemental Fig. S6*G*). In contrast, STT3A and STT3B KO cells significantly attenuated degradation for A2234D Tg. The fraction of degraded A2234D Tg decreased from 50% to 20% 4 h after synthesis (Fig. 5*G*, supplemental Fig. S6*H*). In contrast, STT3A or STT3B KOs did not significantly alter WT Tg degradation rates (supplemental Fig. S6, *G* and *I*). These results suggest that in the case of A2234D, engagement with both OST isoforms is necessary for proper entry into the CANX/CALR lectin folding pathway, resulting in degradation initiated by glycoprotein quality control within the ER.

## Discussion

Most CH-associated Tg missense mutant variants present with very similar phenotypes resulting in inefficient trafficking and loss of secretion. The majority of the CH mutant variants presented here have been reported to be retained within the ER and interact with canonical PN components. Furthermore, L2284P and C1264R have been reported to activate the unfolded protein response (UPR), which acts to adjust ER quality control capacity in response to ER stress (15, 22). UPR activation has also been speculated but not validated for A2234D (40). However, there has been little investigation into (1) identifying molecular mechanisms involved in the inefficient folding and trafficking of these mutant proteins and (2) developing or exploring therapeutic avenues aimed at rescuing the secretion and subsequent hormone production from these mutants (9, 14, 15, 17, 40, 43, 66). Here, we utilized a multiplexed quantitative interactomics platform to describe the PN dependencies of several CH-associated Tg variants with mutations clustering in two different domains of Tg. Our analysis of Tg interactomes reveals common PQC defects that are involved in the loss of Tg secretion, but we also identify unique dependencies that may suggest PQC mechanisms that are specific to individual mutations.

Besides previous documented Tg interactors such as BiP, GRP94, CANX, CALR, and PDIA3, the quantitative interactomics profiling greatly expands our knowledge of additional cochaperones, lectins, trafficking, and degradation factors that influence PQC activity and subsequent Tg processing. Previous work has provided insights on the implications of some of these interactions as BiP overexpression decreases WT Tg secretion (67). Additionally, our analysis identified a number of Hsp40 co-chaperones, which can direct chaperone pathways to assume profolding or prodegradation roles (30, 68). The iterative binding cycles between Tg with BiP and co-chaperones likely results in overall increased retention of Tg and therefore blocks partitioning of Tg to necessary trafficking components (69). PDIA4 has also been identified as a key interactor as it has been shown to bind mutant Tg and form co-aggregates retained within the ER (14). We identified increased interaction between PDIA4 and all secretion-deficient Tg mutants in this study, but we also identified stronger engagement with many other protein disulfide isomerase protein family members and factors involved in oxidative protein folding. Several PDI family members were previously shown to form transient mixed-disulfide–linked intermediates with Tg, highlighting their involvement in Tg folding (14, 15, 50). The increased interactions with these PDIs and additional factors involved in disulfide bond formation may be responsible for intracellular retention and co-aggregation with destabilized Tg variants, which may be mediated through nonresolved mixed-disulfide bond intermediates (14, 58).

Our results show that the CH-associated Tg mutants not only engage many of the chaperoning and oxidative protein folding pathways to a greater extent but also show altered interactions with ERAD and N-glycosylation components that may be responsible for retention and/or aggregation within the ER. Prior work has shown that ERAD of Tg is suppressed upon the inhibition of mannosidase I (MAN1B1) (59). MAN1B1 is known to trim the outermost alpha-1,2-linked mannose residue of the high-mannose ER-associated glycan, followed by subsequent trimming of inner mannose residues, a key step within the glycoprotein ERAD process (70). While we did not identify MAN1B1 in our dataset, other mannosidases of the EDEM family associated with ER stress and UPR activation were identified in the current study (71, 72). These factors exhibited predominantly increased interactions with CH-associated mutants relative to WT Tg, along with accessory proteins such as SEL1L, FOXRED2, OS-9, and other vital glycoprotein ERAD components (31, 52, 56). Considering that mutant Tg variants were not degraded to a greater extent than WT Tg, this increased targeting to ERAD factors seems to be uncoupled from actual proteasomal degradation (73). Ultimately, Tg must be degraded as indicated by prior work and from our CHX and pulse-chase experiments, possibly through a mixture of ERAD activity and autophagy (14, 59). Consistent with this, we identified a number of lysosomal protein factors (Fig. 2*D* and supplemental Fig. S7). Consequently, further investigations into the degradation components and pathways facilitating Tg degradation would be of interest to examine, particularly cross talk and timing between ERAD and autophagy of the endoplasmic reticulum (13, 68, 74, 75, 76).

The identification of altered interactions between CH-associated Tg variants and the components of the OST complex involved in protein N-glycosylation provides new insights into the distinct misprocessing defects for individual CH-associated Tg mutants. While the disruptions in OST complex interactions do not completely explain why these Tg mutants are unable to exit the ER, these changes occur for the most upstream enzymes mediating Tg posttranslational processing, suggesting an important effect. Furthermore, subtle changes in glycosylation patterns could have profound influences on binding of lectin-chaperones, glycan processing enzymes, or lectin-associated oxidoreductases (60, 61, 62, 77).

The changes in PN engagement could be a consequence of changes in Tg secondary and tertiary structure of folding intermediates that then lead to altered engagement with the OST complex. Changes in secondary structure have been loosely predicted for some Tg mutants including extension or reduced stretch of α-helix or β-sheet structure, along with the formation of a β-sheet (78). Interestingly, the cryo-EM structure of Tg showed that the three mutations, A2234D, L2284P, and G2341R, cluster into a small region of the ChEL domain, suggesting that all three mutations could destabilize the structure at a similar location (supplemental Fig. S2*B*). Despite this co-localization, the ChEL mutants displayed several distinct interactome changes, and only A2234D degradation could be rescued by knockout of individual OST isoforms. The same cryo-EM structure also revealed that a glycosylation site at N2013 may play a key role in stabilizing the Tg dimer (38). Investigations into which OST complex is responsible for N2013 glycosylation and how these ChEL domain mutations may change the glycan occupancy would be of particular interest. On the other hand, the C1264R mutation is localized distantly from the ChEL domain in the hinge/flap region, presumably disrupting a local disulfide bond with C1245 and resulting in structurally distinct folding defects (supplemental Fig. S2, *C–D*). Nonetheless, interaction changes with the PN for C1264R are largely similar to the ChEL domain mutants, albeit to a lower magnitude, highlighting that mutations in distinct regions of the protein can produce common PQC deficiency that result in loss of protein secretion. Additionally, disulfide bond formation and N-glycosylation are competing reactions within the ER, further complicating Tg processing (79).

While PQC pathways are unable to facilitate complete folding and secretion of the Tg mutants, folding and processing is clearly attempted before degradation taking place. It may be the case for A2234D Tg, that in the absence of either STT3A or STT3B in the KO cells, proper entry into the lectin-folding cycle is disrupted, ultimately leading to retention within the ER and decreased degradation. The decreased degradation rate may stem from an inability of the PN to recognize A2234D Tg for glycoprotein ERAD because of aberrant glycosylation, or aberrant glycosylation may lead to the preferential aggregation of A2234D Tg, allowing it to escape ERAD (61, 68). Overall, our results highlight that altered proteostasis interactions with Tg variants can have subtle yet significant functional outcomes that are specific to localization and nature of the destabilizing mutation. The cryo-EM structure of human Tg and future structural studies on mutant Tg variants could enable further insights into what structural changes influence the engagement of proteostasis factors (38).

The modulation of PN components or entire pathways has shown recent promise as a therapeutic strategy to combat a number of protein folding diseases (22, 23, 25, 80, 81). By using quantitative multiplexed interactome proteomics, we identified specific PN components that may act as therapeutic targets for rescuing Tg secretion. A similar method has been used to investigate the molecular basis of activating transcription factor 6 (ATF6)-dependent regulation of immunoglobulin light-chain secretion in amyloidosis (45). Methods to pharmacologically target the UPR may be further applicable to rescuing Tg secretion. Many of the CH-associated mutations presented here naturally activate the UPR (9, 15, 40). Therefore, pharmacologic modulation of UPR activity to regulate the abundance of ER proteostasis factors in a coordinated manner could act to restore mutant Tg secretion. In the case of amyloidogenic light-chain proteins, overexpression of UPR-regulated chaperones, in particular BiP and GRP94, was able reduce the secretion of an aggregation-prone protein variant (24, 45). Similar effects were also observed for a model aggregation-prone polyglutamine protein as cytosolic heat shock activation-attenuated intracellular aggregation and cellular toxicity (80). In contrast, the increased surveillance of destabilized Tg variants by chaperoning and oxidative folding pathways are likely directly implicated in the loss of protein secretion. Here, UPR activation potentially further exacerbates the secretion defects for Tg mutants by increasing the abundance of relevant PN components and promoting increased intracellular interactions. Consequently, reducing the engagement between mutant Tg variants and the identified chaperoning, oxidative folding, and ERAD targeting pathways could be a viable strategy to restore mutant Tg secretion (22, 82, 83). Our quantitative proteostasis interactome map forms the framework for the identification of single PN components or entire pathways as viable drug targets geared toward rescuing Tg secretion. Future studies directed at disrupting the individual protein interactions or reducing PN capacity in a coordinated manner through pharmacologic inhibition of UPR signaling pathways could reveal the impact on rescue of CH-associated mutant Tg secretion.

## Data availability

The mass spectrometry proteomics data have been deposited to the ProteomeXchange Consortium via the PRIDE partner repository with the dataset identifier PXD019427. All other necessary data are contained within the manuscript.

## Conflict of interest

Authors declare no competing interests.
