# Supplementary material for: Thyroglobulin Interactome Profiling Defines Altered Proteostasis Topology Associated With Thyroid Dyshormonogenesis
Source: Mol Cell Proteomics. 2020 Dec 8;20:100008. doi: 10.1074/mcp.RA120.002168 (PMC7950113; doi:10.1074/mcp.RA120.002168)
Supplement: Supplemental Material [file mmc1.pdf]

SUPPLEMENTAL INFORMATION FOR:

Thyroglobulin interactome profiling defines altered proteostasis topology associated with thyroid dyshormonogenesis

Madison T. Wright[1], Logan Kouba[2], Lars Plate[1,2,*]

[1]Department of Chemistry, Vanderbilt University, Nashville, TN
[2]Department of Biological Sciences, Vanderbilt University, Nashville, TN

SUPPLEMENTAL INFORMATION FIGURES

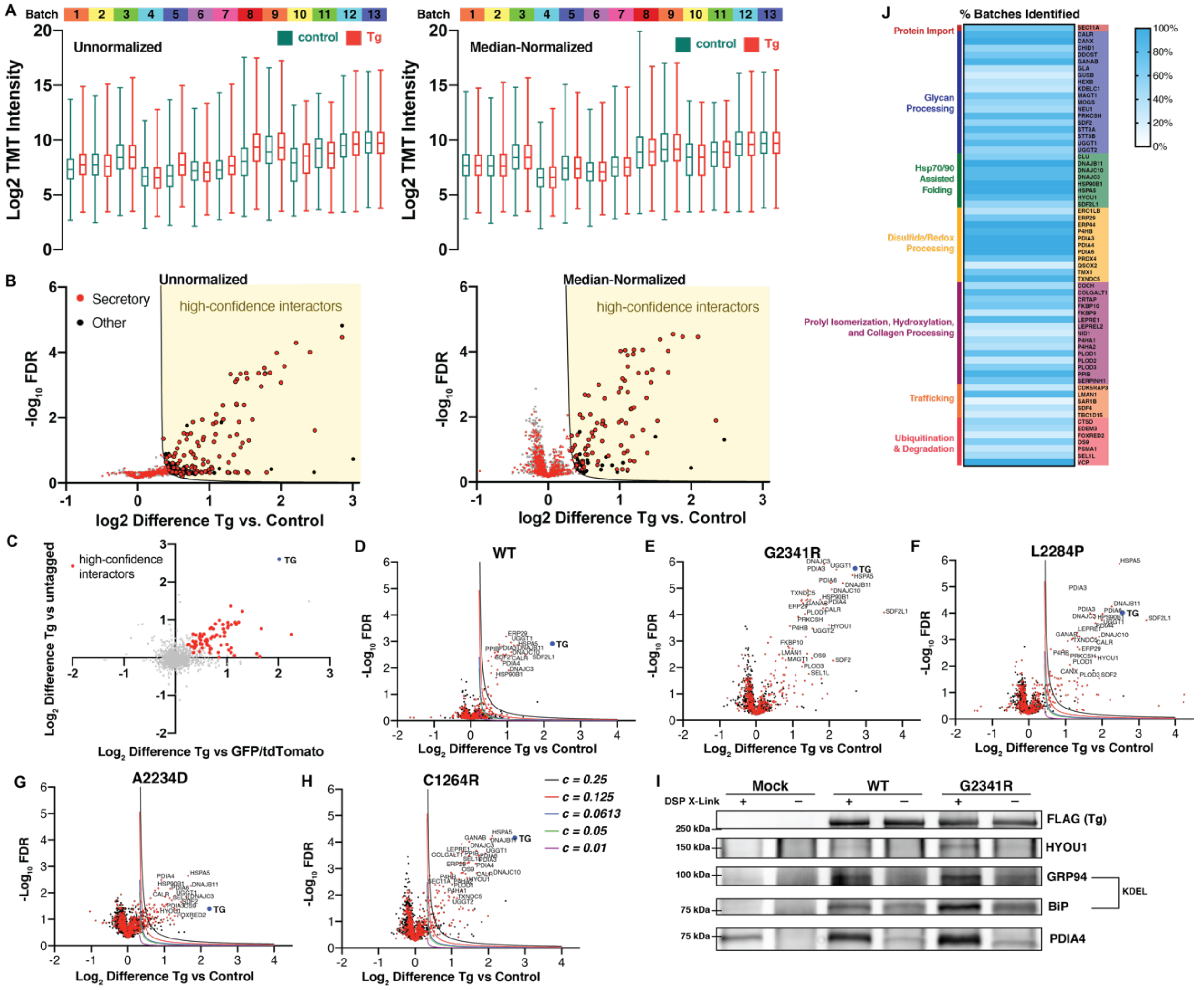

Supplemental Information Figure 1. Processing of quantitative mass spectrometry data to filter high confidence interactors of Tg. A. Distribution of TMT reporter ion intensities of all quantified proteins for the Tg (red) and control channels (green). Raw data unnormalized and transformed data based on median-nomalization is shown. TMT channels that were paired in the same mass spectrometry run are indicated by colored bars on top. Source data is shown in Supplemental Table S3 - Sheets 2-3. **B.** Volcano plot for comparison of protein abundances in Tg vs control channels to identify high-confidence interactors using unnormalized TMT intensities or median-normalized TMT intensities. Plots show the average $\log_2$ difference between combined Tg mutant channels compared to controls vs adjusted p-values (Storey q-values). Median normalization improves the identification of high-confidence thyroglobulin interactors. Source data is shown in Supplemental Table S3 - Sheets 4-5. **C.** Correlation plot comparing enrichment of Tg and confident interactors between untagged WT Tg or GFP/tdTomato controls. The enrichments of Tg and confident interactors are well correlated. As such, untagged WT Tg and

GFP/tdTomato both serve as robust mock controls for AP-MS identification of Tg interactors and were combined in the subsequent analysis. **D-H.** Volcano plots displaying the optimization of cut-off parameters for identification of high-confidence interactors for each Tg variant (WT, G2341R, L2284P, A2234D, C1264R). Plots display the average difference in $\log_2$ TMT reporter ion intensities for proteins between the Tg and control channels vs adjusted p-values (Storey q-values). Cut-offs used to define confident thyroglobulin interactors were optimized using a previously described method, which is further detailed in the experimental procedures (1). Cutoffs were designed in such a way that optimized the identification of secretory pathway components compared to non-specific backgrounds proteins, as described in the Experimental Procedures section. Shown are specific cutoff lines with different curvature parameters *c*. A cutoff value of $c = 0.05$ was selected to define the confident interactors for each Tg variant. Source data is shown in Supplemental Table S3 - Sheets 6-15. Supplemental Table S4 shows the list of confident interactors for each variant, which were then combined into a comprehensive list of Tg interactors for subsequent analysis. **I.** Immunoblot from co-immunoprecipitations with and without DSP crosslinking. $HEK293^{DAX}$ cells were transiently transfected with the mock, WT Tg, or G2341R Tg as indicated, 0.5mM DSP cross-linker or vehicle (DMSO) was added and co-immunoprecipitations (IP) using anti-FLAG antibody-conjugated beads were carried out. Eluted samples were blotted for interaction partners. The addition of crosslinker allows IPs to undergo rigorous washing and still retain interactions with ER proteostasis components that are lost in the absence of crosslinking. **J.** Heatmap indicating the frequency of mass spectrometry batches in which confident Tg interactors were identified.

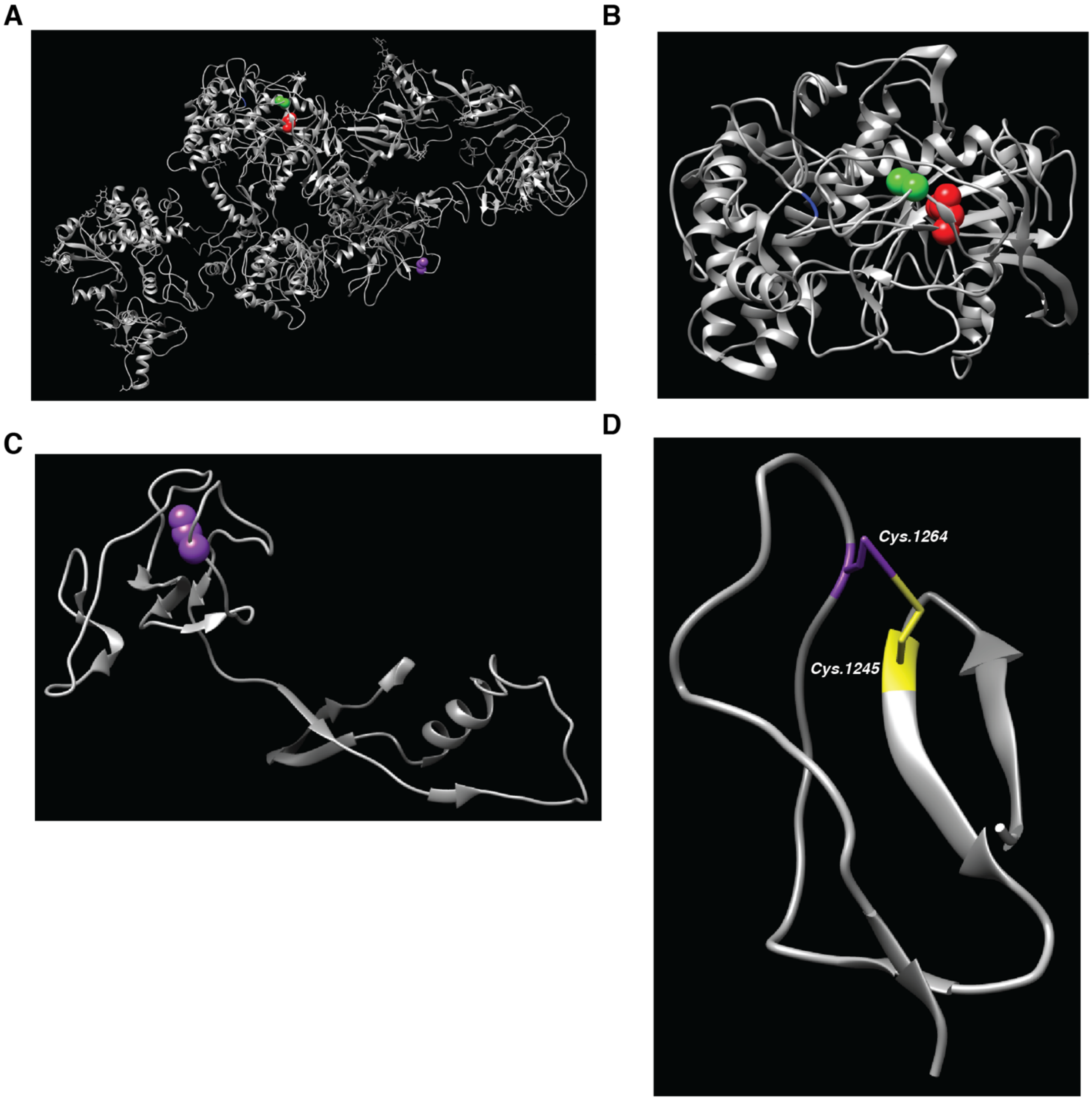

Supplemental Information Figure 2. Structure of thyroglobulin and localization of missense mutations resulting in loss of secretion. A. Cryo-electron microscopy structure of thyroglobulin (PDB ID: 6SCJ). Only one monomer of the Tg dimer is shown for clarity. Missense mutations resulting in folding-incompetent thyroglobulin are annotated: G2341R (Blue), L2284P (Red), A2234D (Green), and C1264R (Purple). **B.** Close up of the Cholinesterase (ChEL)/CTD domain. ChEL/CTD mutations share close proximity with one another. **C.** Close up of the Hinge/Flap region with C1264R mutation. **D.** Close up of the disulfide bridge formed by C1264 and C1245.

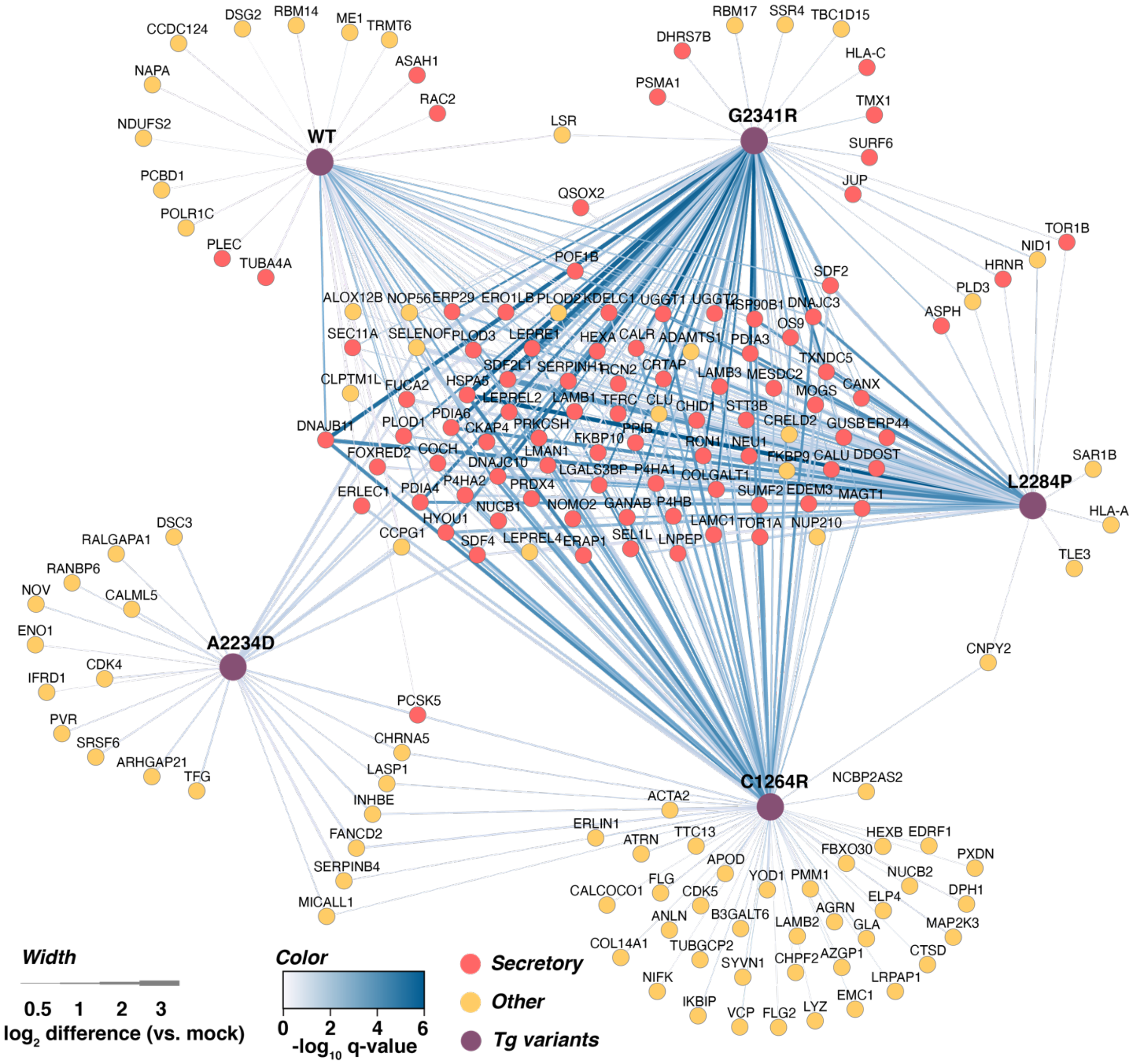

Figure S3. Network plot of Tg interactions with WT and all four mutant variants highlighting shared and unique interactions. Edge thickness represents log2 difference of TMT intensities of the interactors relative to mock controls. Edge color gradient indicates confidence of the interaction (represented by q-value).

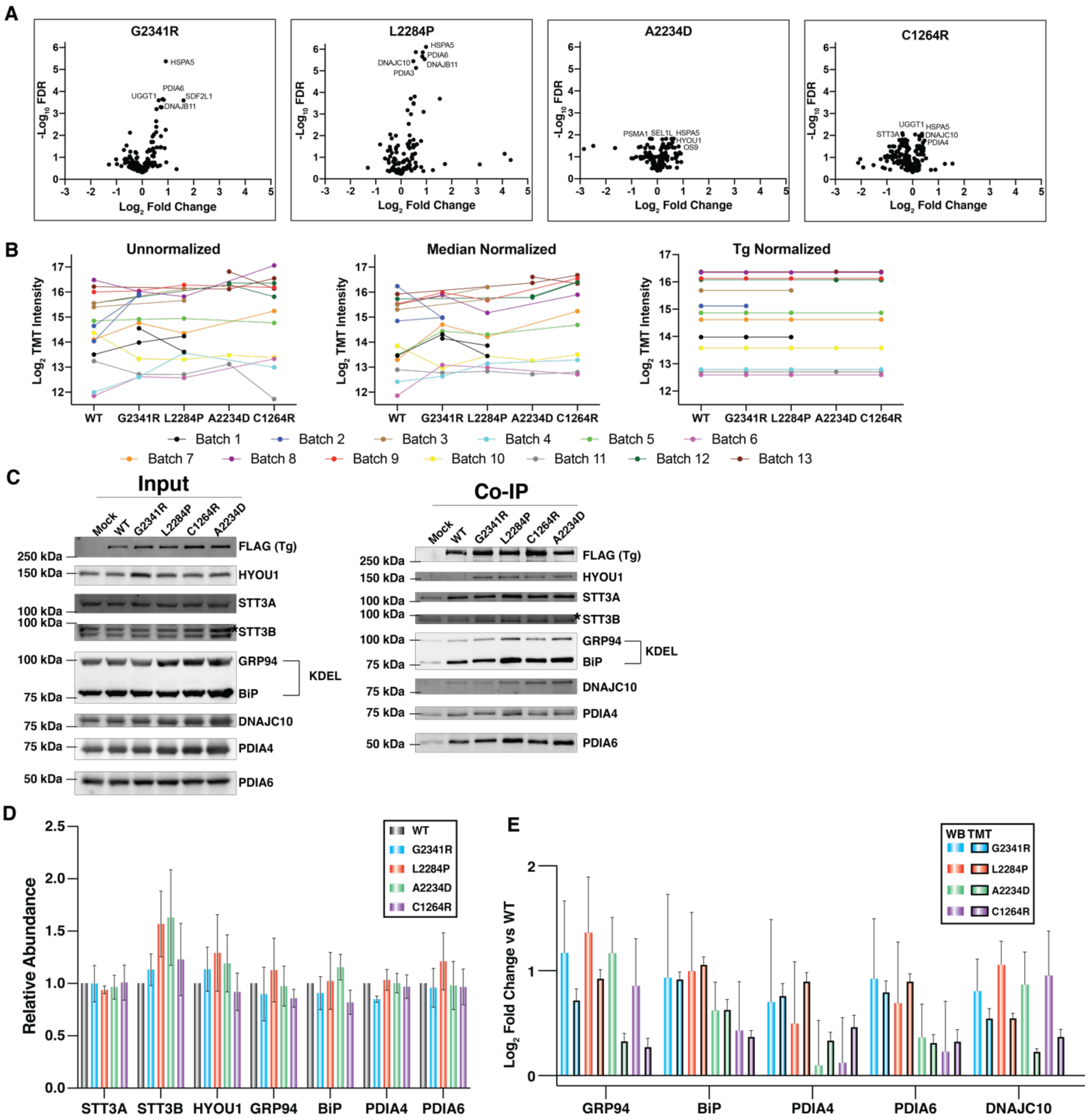

Supplemental Information Figure 4. Comparison of interaction changes between mutant Tg variants and WT Tg. A. Volcano plot showing the interaction changes of high-confidence interactors between individual mutant Tg variants and WT. Plots display the average $\log_2$ difference in TMT reporter ion intensity between the mutant Tg and WT Tg channels for mutant-specific interactors versus the -log10 adjusted p-values (Storey q-values). Source data is shown in Supplemental Table S11. **B.** TMT intensities of Tg variants across the individuals mass spectrometry batches. Shown are unnormalized intensities (left), median-normalized intensities based on total quantified proteins (center), and Tg-normalized intensities (right). **C.** Representative immunoblots from co-immunoprecipitations confirming select

interaction changes between Tg and proteostasis factors. HEK293^{DAX} cells were transiently transfected with the varying Tg constructs or mock as indicated, 0.5mM DSP cross-linker was added to capture transient protein interactions, and co-immunoprecipitations (IP) using anti-FLAG antibody-conjugated beads were carried out identical to the Co-AP experiments for the quantitative proteomics experiments. Lysate inputs are shown as controls. Eluted samples were blotted for interaction partners. **D.** Quantification of the in inputs from the Co-IP experiments from C. Protein intensities were quantified by densitometry in Image Lab (Bio-Rad). Total protein amounts of Tg interactors remain largely unchanged in cells expressing Tg indicating that the interaction differences are not due to changes in protein abundances. Error bars show SEM from n = 2-3 experiments. **E.** Quantification of the Co-IP experiments from C. Protein intensities were quantified by densitometry in Image Lab (Bio-Rad) and protein amounts of interactors in the Co-IP samples were normalized to abundances of Tg variants (FLAG). Error bars show SEM from n = 3 experiments. For comparison, interaction fold changes from TMT-based quantitative AP-MS are shown in bold outlines. Increased interactions with chaperoning and oxidative folding pathways were confirmed.

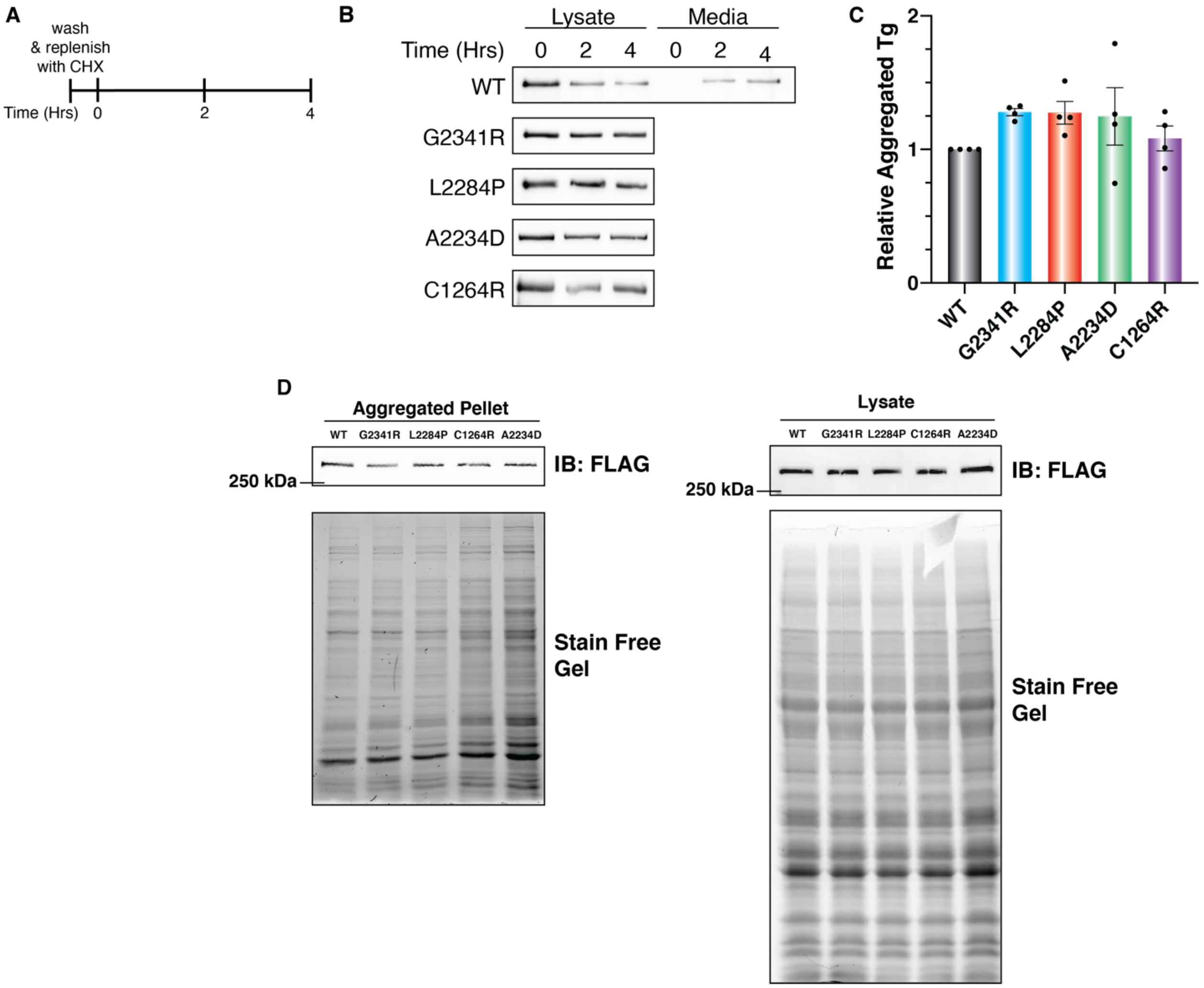

Supplemental Information Figure 5. Cycloheximide chase and aggregation analysis of WT and CH-associated Tg. A. Treatment scheme for cycloheximide (CHX) chase assay. **B.** Representative immunoblots from CHX chase assays in Fig. 4E to measure degradation rates of Tg variants. FLAG-tagged Tg variants were transiently transfected into HEK293T cells and treated as outlined in A. Tg from media (WT only) or lysate samples was immunoprecipitated using anti-FLAG antibody beads, resolved by SDS-PAGE followed by immunoblotting. **C.** Relative quantification of Tg aggregation. Proteins in the insoluble pellet after lysis were resolubilized in urea, resolved by SDS-PAGE followed by immunoblotting. While all Tg constructs aggregate within the cell, mutant Tg aggregation is not signicantly increased compared to WT. **D.** Representative immunoblots and corresponding gels from solubilized aggregates and cell lysates.

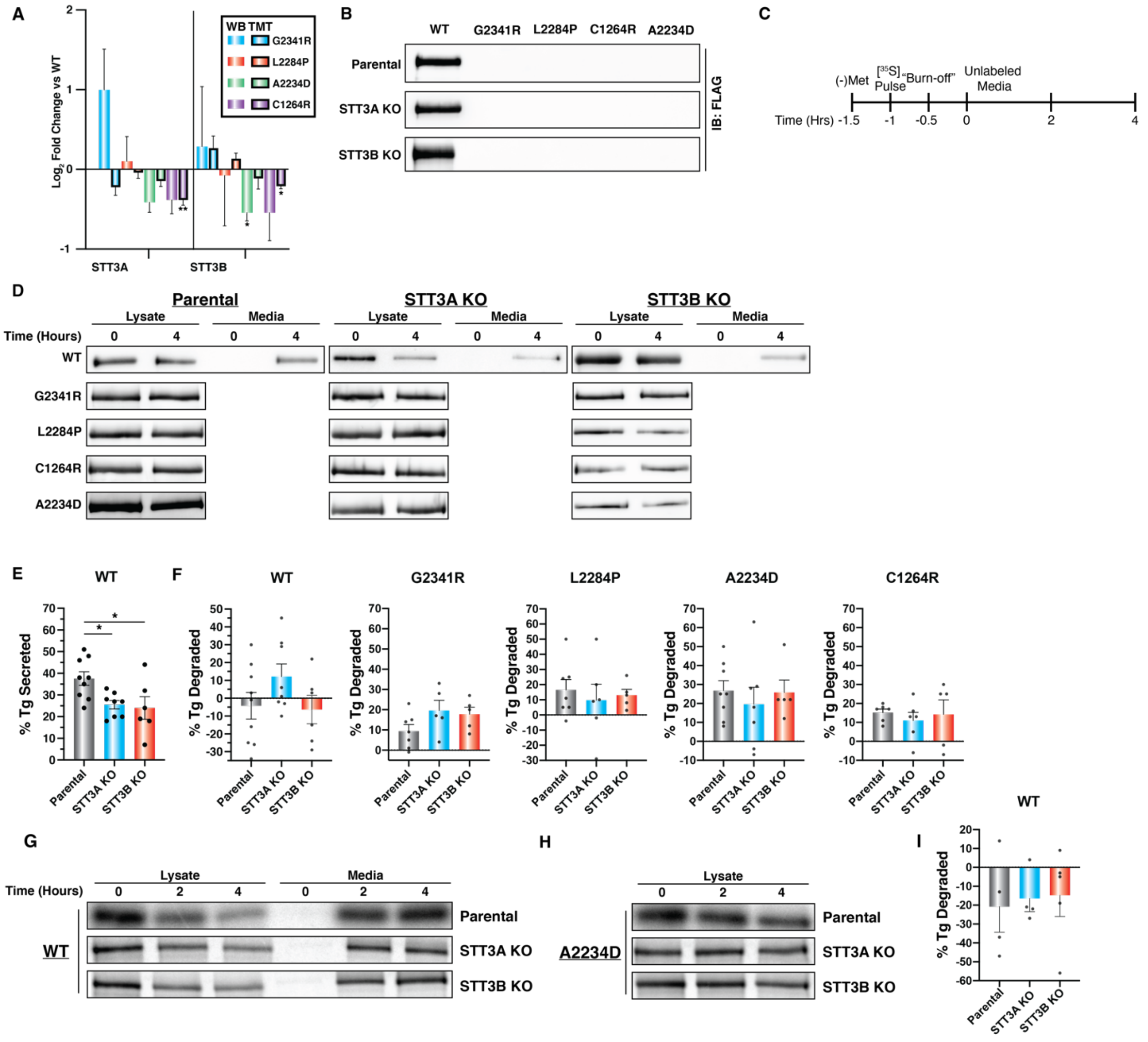

Supplemental Information Figure 6. Cycloheximide chase and pulse-chase assays to measure Tg processing in STT3A and STT3B KO cells. A. Confirmation of decreased protein interactions with the OST subunits STT3A and STT3B by Co-IP followed by immunoblots. Error bars shows SEM from n = 3 experiments. For comparison, interaction fold changes from TMT-based quantitative AP-MS are shown in bold outlines. Student's parametric t-test was used to determine significant changes for immunoblot quantification. Student's parametric t-test with Storey multiple testing corrections was used to determine significant changes for TMT quantification. *: $q < 0.05$, **: $q < 0.005$. **B**. Immunoblots of immunoprecipitated media samples from cells transiently transfected with respective FLAG-tagged Tg plasmid constructs. Neither STT3A or STT3B knockdown impair WT Tg secretion or rescue mutant Tg secretion. **C.** Representative immunoblots from CHX chase assays performed using STT3A and STT3B KO cells to investigate the role of the OST complex isoforms on Tg processing. FLAG-tagged Tg

variants were transiently transfected into parental HEK293T or STT3A or STT3B KO cell lines and treated as outlined in A. Tg from media (WT only) or lysate samples was immunoprecipitated using anti-FLAG antibody beads, resolved by SDS-PAGE followed by immunoblotting. Tg protein bands were quantified by densitometry in Image Lab (Bio-Rad). **D**. Treatment scheme for ^{35}S-metabolic labeling and pulse-chase assay. **E.** Plot showing the quantified changes in WT Tg secretion for WT Tg as measured by CHX chase assay in parental, STT3A or STT3B KO cells. % Tg secreted was calculated as $Tg_{media,\,4h} / \left(Tg_{lysate,\,0h} + Tg_{media,\,0h}\right)$. Error bars display SEM of 6-9 biological replicates. Student's parametric t test was used to determine significant changes in Tg secretion and p values are indicated. **F.** Plots showing the quantified changes in Tg degradation as measured by CHX chase assay in parental, STT3A or STT3B KO cells. % Tg degraded was calculated as 1 - $(Tg_{lysate,\,4h} / Tg_{lysate,\,0h})$ for Tg mutants and 1 - $(Tg_{lysate,\,4h} + Tg_{media,\,4h})$ for WT. Error bars display SEM of 5-9 biological replicates. Student's parametric t test was used to determine significant changes in Tg degradation and p values are indicated. **G-H.** Representative autoradiograms of ^{35}S-pulse chase experiment to measure Tg secretion and degradation. FLAG-tagged Tg variants were transiently transfected into parental HEK293T or STT3A or STT3B KO cell lines and treated as outlined in B. Tg from media (WT only) or lysate samples was immunoprecipitated using anti-FLAG antibody beads, resolved by SDS-PAGE followed by autoradiography. ^{35}S-labeled Tg protein bands were quantified by densitometry in Image Lab (Bio-Rad). **I.** Plot showing the quantified changes in WT Tg degradation as measured by ^{35}S-pulse chase experiments. Error bars display SEM of 4-5 biological replicates. Student's parametric t test was used to determine significant changes in Tg secretion and p values are indicated.

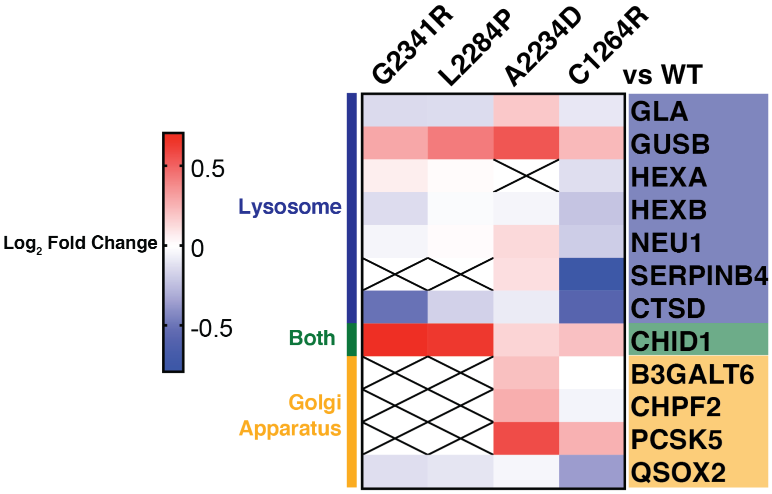

Supplemental Information Figure 7. Heatmap displaying altered engagement of lysosomal and Golgi related proteostasis components for mutant Tg variants compared to WT Tg.

SUPPLEMENTAL INFORMATION TABLES

(Tables are included as separate .xlxs or .docx files)

Supplemental Information Table S1. Table detailing the primers used to perform site-directed mutagenesis on the FLAG tagged WT Tg-pcDNA3.1 plasmid, as described in the Materials and Methods section.

Supplemental Information Table S2. Table detailing the layout of mock, WT or mutant Tg co-immunoprecipitation replicate samples across the different mass spectrometry batches. The TMT channels for each sample, as well as the instrument used for each batch are indicated.

Supplemental Information Table S3. Source data for Supplemental Fig. S1. Sheets 1 - 5 contain the data used to compare unnormalized vs median normalized TMT intensities to generate plots in Supplemental Fig. S1A-B. Median normalization results yield better identification of confident Tg interactors. Sheets 6-15 contain the data used to create the plots in Supplemental Fig. S1D-H. True Tg interactors were further delineated as detailed in Supplemental Table S4 and the Experimental Procedures section.

Supplemental Information Table S4. After optimizing normalization methods and identifying optimal cutoffs, Tg interactors were identified for individual constructs. Tg interactors found from individual constructs were combined to give a comprehensive list of the Tg interactome. This subsequent list was then used for further data analysis.

Supplemental Information Table S5. Gene Ontology of cellular component terms used to delineate identified proteins that were found to be components of the secretory pathway. Further grouping of high-confidence interactors into proteostasis sub-pathways used in Fig. 3 is indicated.

Supplemental Information Table S6. Raw, unprocessed, protein identification and quantification data from AP-MS experiments used for interactome characterization. Spectra and result files are available via ProteomeXchange under identifier PXD019427.

Supplemental Information Table S7. Peptide identification data from AP-MS experiments used for interactome characterization. Spectra and result files are available via ProteomeXchange under identifier PXD019427.

Supplemental Information Table S8. Information on biological significance and references for mutant Tg variants.

Supplemental Information Table S9. Comparison of Tg interactors identified in BioPlex, BioGRID and STRING with our dataset.

Supplemental Information Table S10. Source data for Figure 3. Log_2 fold change of TMT intensities for interactors comparing CH-associated mutant Tg constructs vs WT Tg. Components sorted by 2018 GO Biological Process terms. For dot plots, individual Tg interactors are plotted together based on GO terms, with each dot corresponding to an individual interactor.

Supplemental Information Table S11. Source data for Supplemental Figure S4A. Log_2 fold change of TMT intensities and FDR estimation for interactors comparing CH-associated mutant Tg constructs vs WT Tg.

SUPPLEMENTAL INFORMATION REFERENCES

1. Keilhauer, E. C., Hein, M. Y., and Mann, M. (2015) Accurate Protein Complex Retrieval by Affinity Enrichment Mass Spectrometry (AE-MS) Rather than Affinity Purification Mass Spectrometry (AP-MS). *Mol. Cell. Proteomics* 14, 120–135
