# Supplementary material for: Thyroglobulin Interactome Profiling Defines Altered Proteostasis Topology Associated With Thyroid Dyshormonogenesis
Source: Mol Cell Proteomics. 2020 Dec 8;20:100008. doi: 10.1074/mcp.RA120.002168 (PMC7950113; doi:10.1074/mcp.RA120.002168)
Supplement: Supplemental Table S1 [file mmc2.docx]

| Oligo Nucleotide Construct | Sequence | |
| --- | --- | --- |
| G2341R Site-Directed Mutagenesis Forward Primer | | GAGTGGGTGTCTTCCGCTTCCTGAGTTC |
| G2341R Site-Directed Mutagenesis Reverse Primer | | GAACTCAGGAAGCGGAAGACACCCACTC |
| L2284P Site-Directed Mutagenesis Forward Primer | GAAGATTGTTTGTATCCCAATGTGTTCATCCCTC | |
| L2284P Site-Directed Mutagenesis Reverse Primer | GAGGGATGAACACATTGGGATACAAACAATCTTC | |
| A2234D Site-Directed Mutagenesis Forward Primer | AGTTCCATATGaTGCCCCGCCCC | |
| A2234D Site-Directed Mutagenesis Reverse Primer | GAAGCAAGTGGACCAGTTCCTTGG | |
| C1264R Site-Directed Mutagenesis Forward Primer | CAGGGCCATTGATACGTAGCCTGGAGAGC | |
| C1264R Site-Directed Mutagenesis Reverse Primer | GCTCTCCAGGCTACGTATCAATGGCCCTG | |
| D2769X (Untagged WT) Site-Directed Mutagenesis Forward Primer | GCTCTAAGACCTACAGCAAGTGATACAAGGATGACGACGATAAG | |
| D2769X (Untagged WT) Site-Directed Mutagenesis Reverse Primer | CTTATCGTCGTCATCCTTGTATCACTTGCTGTAGGTCTTAGAGC | |
