# Supplementary material for: Thyroglobulin Interactome Profiling Defines Altered Proteostasis Topology Associated With Thyroid Dyshormonogenesis
Source: Mol Cell Proteomics. 2020 Dec 8;20:100008. doi: 10.1074/mcp.RA120.002168 (PMC7950113; doi:10.1074/mcp.RA120.002168)
Supplement: Supplemental Table S2 [file mmc3.docx]

| Instrument | Batch Number | TMT Labeling Scheme | | | | | |
| --- | --- | --- | --- | --- | --- | --- | --- |
|  |  | 126 | 127 | 128 | 129 | 130 | 131 |
| Q-Exactive HF | 1 | WT | GFP | G2341R | G2341R | L2284P | L2284P |
|  | 2 | tdTomato | tdTomato | WT | WT | G2341R | G2341R |
|  | 3 | tdTomato | tdTomato | WT | WT | L2284P | L2284P |
|  | 4 | tdTomato | tdTomato | WT | G2341R | L2284P | C1264R |
|  | 5 | tdTomato | tdTomato | WT | G2341R | L2284P | C1264R |
|  | 6 | GFP | GFP | WT | G2341R | L2284P | C1264R |
|  | 7 | tdTomato | tdTomato | WT | G2341R | L2284P | C1264R |
|  | 8 | GFP | Untagged | WT | G2341R | L2284P | C1264R |
|  | 9 | tdTomato | Untagged | WT | G2341R | L2284P | C1264R |
|  | 10 | Untagged | WT | G2341R | L2284P | C1264R | A2234D |
|  | 11 | Untagged | WT | G2341R | L2284P | C1264R | A2234D |
| Exploris 480 | 12 | Untagged | WT | C1264R | C1264R | A2234D | A2234D |
|  | 13 | Untagged | WT | C1264R | C1264R | A2234D | A2234D |
