# Supplementary material for: Thyroglobulin Interactome Profiling Defines Altered Proteostasis Topology Associated With Thyroid Dyshormonogenesis
Source: Mol Cell Proteomics. 2020 Dec 8;20:100008. doi: 10.1074/mcp.RA120.002168 (PMC7950113; doi:10.1074/mcp.RA120.002168)
Supplement: Supplemental Table S8 [file mmc9.docx]

| Missense Mutation | Tg Region | Biological Significance | References (First Author, Year, Ref.) |
| --- | --- | --- | --- |
| G2341R | ChEL Domain | Novel mutation first presented here, exhibits trafficking phenotype similar to that of L2284P Tg | N/A |
| L2284P | ChEL Domain | Originally discovered in cog/cog mice resulting in congenital hypothyroidism with goiter; expression known to activate the unfolded protein response (UPR); thyrocytes shown to survive under chronic stress due to expression of the misfolded construct; protein-protein interaction partners and associated changes are uncharacterized | Kim, 1996, 9.  Medeiros-Neto, 1996, 10.  Kim, 1998, 39.  Park, 2004, 16. |
| A2234D | ChEL Domain | Originally discovered in human patients resulting in congenital hypothyroidism with goiter; low levels of secretion may be present to produce sufficient thyroid hormones for proper embryonic development, protein-protein interaction partners and associated changes are uncharacterized | Caputo, 2007, 40.  Pardo, 2008, 78.  Pardo, 2009, 43. |
| C1264R | Hinge/Flap Region | Originally discovered in human patients resulting in congenital hypothyroidism with goiter; expression linked to the activation of the unfolded protein response (UPR); expression of this construct in patients is correlated with increased D2 activity (D2, deiodinase enzyme responsible for the conversion of T4 to T3); | Hishinuma, 1999, 17.  Kanou, 2007, 66. |
