# Supplementary material for: Thyroglobulin Interactome Profiling Defines Altered Proteostasis Topology Associated With Thyroid Dyshormonogenesis
Source: Mol Cell Proteomics. 2020 Dec 8;20:100008. doi: 10.1074/mcp.RA120.002168 (PMC7950113; doi:10.1074/mcp.RA120.002168)
Supplement: Supplemental Table S9 [file mmc10.docx]

| Tg Interactor | Bioplex* | BioGRID | STRING | Wright et al. |
| --- | --- | --- | --- | --- |
| HSPA5 | N/A | X |  | X |
| P4HB | N/A | X |  | X |
| LRP2 | N/A |  | X |  |
| ASGR1 | N/A |  | X |  |
| TPO | N/A |  | X |  |
| PAX8 | N/A |  | X |  |
| NKX2-1 | N/A |  | X |  |

* Tg not present within the Bioplex database
